# Interference in the processing of adjunct control

**DOI:** 10.3389/fpsyg.2015.01346

**Published:** 2015-09-08

**Authors:** Dan Parker, Sol Lago, Colin Phillips

**Affiliations:** ^1^Linguistics Program, Department of English, College of William and MaryWilliamsburg, VA, USA; ^2^Department of Linguistics, University of MarylandCollege Park, MD, USA; ^3^Language Science Center, University of MarylandCollege Park, MD, USA

**Keywords:** adjunct control, anaphora, agreement, sentence processing, memory retrieval

## Abstract

Recent research on the memory operations used in language comprehension has revealed a selective profile of interference effects during memory retrieval. Dependencies such as subject–verb agreement show strong facilitatory interference effects from structurally inappropriate but feature-matching distractors, leading to illusions of grammaticality (Pearlmutter et al., 1999; Wagers et al., 2009; Dillon et al., 2013). In contrast, dependencies involving reflexive anaphors are generally immune to interference effects (Sturt, 2003; Xiang et al., 2009; Dillon et al., 2013). This contrast has led to the proposal that all anaphors that are subject to structural constraints are immune to facilitatory interference. Here we use an animacy manipulation to examine whether adjunct control dependencies, which involve an interpreted anaphoric relation between a null subject and its licensor, are also immune to facilitatory interference effects. Our results show reliable facilitatory interference in the processing of adjunct control dependencies, which challenges the generalization that anaphoric dependencies as a class are immune to such effects. To account for the contrast between adjunct control and reflexive dependencies, we suggest that variability within anaphora could reflect either an inherent primacy of animacy cues in retrieval processes, or differential degrees of match between potential licensors and the retrieval probe.

## Introduction

Linguistic dependencies are subject to diverse structural and morphological constraints. Recent studies have examined how these constraints are applied in real-time comprehension in order to gain a better understanding of how we mentally encode and navigate linguistic representations. A comparison of the findings across studies shows a mixed profile of successes and failures of real-time constraint application: some constraints on dependency formation are accurately applied, whereas others are susceptible to errors. The reasons for these failures remain poorly understood, but the mixed profile of constraint application has been argued to reflect the way in which different linguistic processes engage memory retrieval mechanisms (Lewis and Vasishth, 2005; Vasishth et al., 2008; Wagers et al., 2009; Phillips et al., 2011; Lewis and Phillips, 2015).

In this paper, we focus on a specific type of memory retrieval error that leads to an effect called ‘facilitatory interference’ (also known as ‘intrusion’ or ‘attraction’). Facilitatory interference arises when a structurally inappropriate but feature matching item facilitates the processing of an ill-formed linguistic dependency. This eased processing can trigger ‘illusions of grammaticality,’ which have been argued to reflect limitations of the memory retrieval mechanisms used to implement linguistic constraints (Vasishth et al., 2008; Wagers, 2008). Such effects have been reported for subject–verb agreement and negative polarity item processing (Clifton et al., 1999; Pearlmutter et al., 1999; Drenhaus et al., 2005; Vasishth et al., 2008; Staub, 2009, 2010; Wagers et al., 2009; Xiang et al., 2009; Dillon et al., 2013; Tanner et al., 2014; Tucker et al., 2015). For instance, Wagers et al. (2009) used self-paced reading and speeded acceptability judgments to investigate interference effects in the comprehension of subject–verb agreement dependencies like those in (1). They varied the presence of a plural distractor noun in grammatical and ungrammatical sentences.

(1) a.  The key to the *cell(s)* unsurprisingly was rusty from many years of disuse.
b.  ^∗^The key to the *cell(s)* unsurprisingly were rusty from many years of disuse.

In grammatical sentences like (1a), Wagers et al. (2009) found that the plural number of the structurally inappropriate noun *cells* did not impact acceptability judgments or reading times after the verb, relative to the singular noun condition. However, in ungrammatical sentences like (1b) the presence of the plural distractor *cells* increased rates of acceptance and facilitated reading times after the verb, relative to the no distractor condition, giving rise to an illusion of grammaticality.

The profile of facilitatory interference effects in sentences like (1) provides important evidence about the source of these effects in comprehension. First, immunity to distractors in grammatical sentences suggests that facilitatory interference effects do not reflect misrepresentation of the subject number or the use of “good enough” representations (e.g., Ferreira and Patson, 2007). Misrepresentation of the subject phrase would lead comprehenders to misperceive grammatical sentences like (1a) as ungrammatical, triggering an ‘illusion of ungrammaticality,’ which rarely occurs. Second, if illusions of grammaticality are not due to problems in the representation of the subject, then facilitatory interference effects might instead be due to properties of the retrieval mechanisms used to resolve linguistic dependencies. For instance, under a view where both structural and morphological constraints guide memory access, facilitatory interference effects could reflect failure to apply structural constraints during retrieval, or they could reflect the outcome of a competition between structural and morphological constraints. Crucially, the finding that comprehenders are not misled by structurally inappropriate items in grammatical sentences provides good evidence that structural constraints are actively used to guide retrieval, and suggests that facilitatory interference reflects competing structural and morphological information^1^.

Wagers et al. (2009) argued that facilitatory interference in subject–verb agreement is a consequence of competing constraints. Under their account, encountering the verb *were* in (1b) triggers a retrieval that probes previous items in memory to recover a noun phrase that is both the subject of the sentence and has plural number. In ungrammatical sentences, neither the target nor the distractor is a perfect match to the requirements of the verb, and the competition between the structural and morphological constraints is relatively even: the true subject is in the appropriate structural position, but it is not plural, and the distractor is plural, but it is in a structurally inappropriate position. On a significant portion of trials, the structurally inappropriate distractor is incorrectly retrieved, which facilitates processing of the ungrammatical verb and triggers an illusion of grammaticality. In grammatical sentences, by contrast, there is no competition between the structural and morphological constraints, and the full matching subject is almost always retrieved, as it easily out-competes a non-matching distractor^2^.

Facilitatory interference is robust for subject–verb agreement, but not all linguistic dependencies are susceptible to it. For example, Dillon et al. (2013) directly compared the processing of subject–verb agreement and reflexive-antecedent dependencies using closely matched sentences like those in (2).

(2) a.  The new executive who oversaw the middle *manager(s)* apparently doubted himself/^∗^themselves on most major decisions.
b.  The new executive who oversaw the middle *manager(s)* apparently was/^∗^ were dishonest about the company’s profits.

Dillon et al. (2013) found that subject–verb agreement was susceptible to facilitatory interference from structurally inappropriate distractors (e.g., *managers*), but reflexive-antecedent dependencies were not. These findings are consistent with a growing number of studies that have concluded that direct object reflexives resist facilitatory interference (Nicol and Swinney, 1989; Clifton et al., 1999; Kennison and Trofe, 2003; Sturt, 2003; Xiang et al., 2009; Clackson et al., 2011; Jäger et al., 2015a). Specifically, these studies have found that structurally inappropriate distractors either do not impact the processing of the direct object reflexives or cause increased processing difficulty. The contrast between subject–verb agreement and reflexives is striking since retrieval for both dependencies targets the same structural position, i.e., the subject of the local clause. These findings are important because they cast doubt upon the claim that all linguistic dependencies are uniformly resolved using an error-prone retrieval mechanism, as suggested in previous research (McElree, 2000; McElree et al., 2003; Lewis and Vasishth, 2005).

The puzzle of why reflexives and subject–verb agreement show contrasting profiles with respect to facilitatory interference remains unresolved. One explanation that is often suggested is that the contrast may reflect differences in the interpretive status of reflexives vs. agreement (see Dillon, 2011, for discussion). Reflexive licensing involves constructing an interpreted anaphoric dependency since the meaning of the reflexive depends on the semantic properties of its antecedent. By contrast, subject–verb agreement licensing might involve a morphological process without interpretive consequences (e.g., Lau et al., 2008; but cf. Patson and Husband, 2015). However, it is unclear why the interpretive status of a dependency should determine its susceptibility to facilitatory interference. One possibility is that all interpreted anaphoric dependencies that are subject to syntactic constraints might engage a more conservative retrieval strategy to avoid misinterpretation and interference from structurally inappropriate items. Under this hypothesis, reflexives and agreement could engage qualitatively different retrieval mechanisms, or use distinct sets of retrieval cues to access the local subject. For example, reflexive licensing might engage the same retrieval mechanism as agreement, but might only use structural retrieval cues, implementing morphological constraints only as a post-retrieval check (Dillon, 2011).

In this paper, we do not solve the problem of why reflexives and subject–verb agreement show differential susceptibility to facilitatory interference effects. Instead, we address a critical part of the puzzle by focusing on the status of anaphors and their reported immunity to such effects. Specifically, we test the hypothesis that all anaphoric dependencies that are subject to structural constraints avoid facilitatory interference during real-time comprehension. Our results challenge this hypothesis by showing that adjunct control dependencies, which involve an interpreted anaphoric relation between a null subject and its licensor, are susceptible to facilitatory interference. We then investigate the source of facilitatory interference in adjunct control dependencies, and conclude with a discussion of why anaphoric dependencies should vary with respect to facilitatory interference effects.

### Adjunct Control Dependencies

In this paper, we focus on temporal adjunct control constructions like those in (3), which involve a phonetically null anaphoric subject (represented as ∅)^3^. Like reflexives, null subjects must establish a structural, item-to-item dependency with a licensor to receive an interpretation. Specifically, null subjects in temporal adjunct control structures are licensed by the subject of the immediately higher clause. For instance, in (3a,b), the phonetically null subject of the adjunct clause ∅ receives its interpretation from the subject of the immediately higher clause *the little girl*, i.e., it is the little girl who played in the yard.

(3) a.  The mother said [that *the little girl* fell asleep (after ∅ playing in the yard)].
b.  *The little girl* talked to her mother (after ∅ playing in the yard).

However, there are several differences between reflexives and null subjects that might impact their susceptibility to facilitatory interference. For example, reflexives are licensed by the subject of the local clause, whereas null subjects in temporal adjunct control structures are licensed by the subject of the immediately higher clause. Another difference is that retrieval for reflexive licensing is triggered by an independent anaphoric element, whereas retrieval for null subject licensing is triggered by a gerundive verb preceded by a subordinator (e.g., “*after playing”*). Lastly, unlike reflexives, null subjects do not require overt gender or number agreement with a licensor. Instead, null subject licensing in adjunct control structures has been argued to be subject to an animacy constraint. For example, Kawasaki (1993) reported that adjunct control structures are judged to be more acceptable with animate licensors than inanimate licensors (4a vs. 4b; see Landau, 2001, for supporting judgments). The preference for an animate subject does not appear to be a general property of embedded clauses or a consequence of lexical verb biases, since the acceptability contrast between (4a) and (4b) is neutralized when the licensors are the overt subjects of the verb, as in (5).

(4) a.  The doctor was certified after ∅ debunking the hypothesis.
b.  The discovery was certified after ∅ debunking the hypothesis.

(5) a.  The journalist was surprised that the doctor debunked the hypothesis.
b.  The journalist was surprised that the discovery debunked the hypothesis.

The current study contributes to a growing body of research on the processing of control (e.g., Kwon and Sturt, 2014; Sturt and Kwon, 2015) by using the animacy preference for adjunct control structures to probe for facilitatory interference during real-time dependency formation. Animacy features are promising candidates to test for interference effects, as they have been shown to be used in memory retrieval during processing of various linguistic dependencies, including thematic binding and reflexive licensing (e.g., Van Dyke and Lewis, 2003, 2007; Van Dyke and McElree, 2006, 2011; Jäger et al., 2015b). Specifically, we contrast two hypotheses about the nature of retrieval for anaphoric dependencies. Under a view that posits that all anaphoric dependencies are immune to facilitatory interference, null subjects in temporal adjunct control structures should pattern like reflexives and show no susceptibility to facilitatory interference during retrieval for a licensor. In contrast, if anaphoric dependencies do not behave homogenously, then null subject licensing might show facilitatory interference effects similar to those observed for subject–verb agreement.

We report the results from three experiments. In Experiment 1 (untimed acceptability ratings) we confirmed the animacy constraint on null subject licensing. In Experiments 2 and 3 (self-paced reading), we directly compared the comprehension of null subjects and subject–verb agreement, and found that null subjects show a facilitatory interference profile that is qualitatively similar to the profile observed for agreement. These results imply that not all anaphoric dependencies resist facilitatory interference, and suggest that differences in interpretative status cannot be uniquely responsible for the contrasting interference profiles reported for agreement and reflexives in previous studies.

## Experiment 1

Experiment 1 used untimed acceptability ratings to confirm that temporal adjunct control sentences are more acceptable with animate than inanimate licensors, and that the preference for animate licensors is specific to adjunct control constructions, rather than a general property of embedded clauses or lexical verb biases (Kawasaki, 1993).

### Participants

Twenty-four participants were recruited using Amazon’s Mechanical Turk web-service^4^ All participants in this and the following experiments provided informed consent. Experiment 1 lasted approximately 10 min, and participants were compensated $2.

### Materials

Twenty-four sets of items like those in (4–5) were constructed. Two experimental factors were manipulated: ANIMACY of the main clause subject (animate vs. inanimate) and CONSTRUCTION (adjunct control vs. overt subject). The 24 item sets were distributed across four lists in a Latin Square design. Within each list, the 24 target sentences were combined with 48 filler sentences of similar length and complexity, for a total of 72 sentences. The ratio of grammatical to ungrammatical sentences was 1:1, including the inanimate adjunct control sentences as ungrammatical. The ungrammatical filler sentences involved subject–verb agreement errors, unlicensed verbal morphology, and selectional restriction violations.

### Procedure

Sentences were presented using Ibex (Alex Drummond^5^). Participants were instructed to rate the acceptability of the sentences along a 7-point Likert scale (‘7’ = most acceptable, ‘1’ = least acceptable), according to their perceived acceptability in informal, colloquial speech. Participants could take as much time as needed to rate each sentence, as long as they finished the experiment within the 30 min restriction imposed by the Mechanical Turk session. Each sentence was displayed in its entirety on the screen along with the rating scale. Participants could click boxes to enter their rating or use a numerical keypad. The order of presentation was randomized for each participant.

### Data Analysis

Data were analyzed using linear mixed-effects models, with fixed factors for experimental manipulations and their interaction. Models were estimated using the *lme4* package (Bates et al., 2011) in the R software environment (R Development Core Team, 2014). Experimental fixed effects and their interaction were set up using orthogonal contrast coding, and items and participants were crossed as random effects (following Baayen et al., 2008; Bates et al., 2011). To determine whether inclusion of random slopes was necessary, we compared a model that included random by-participant and by-item intercepts with a model that included a fully specified (i.e., maximal) random effects structure with random intercepts and slopes for all random effects and their interaction by-item and by-participant (Baayen et al., 2008; Barr et al., 2013). A log-likelihood ratio test revealed that the maximal model provided a better fit to the data [χ^2^_(2)_ = 67.36, *p* < 0.001]. Therefore, we adopted the maximal model. For all statistical analyses reported in this paper, an effect was considered significant if its absolute *t*-value was greater than 2 (Gelman and Hill, 2007).

### Results

**FIGURE 1:**
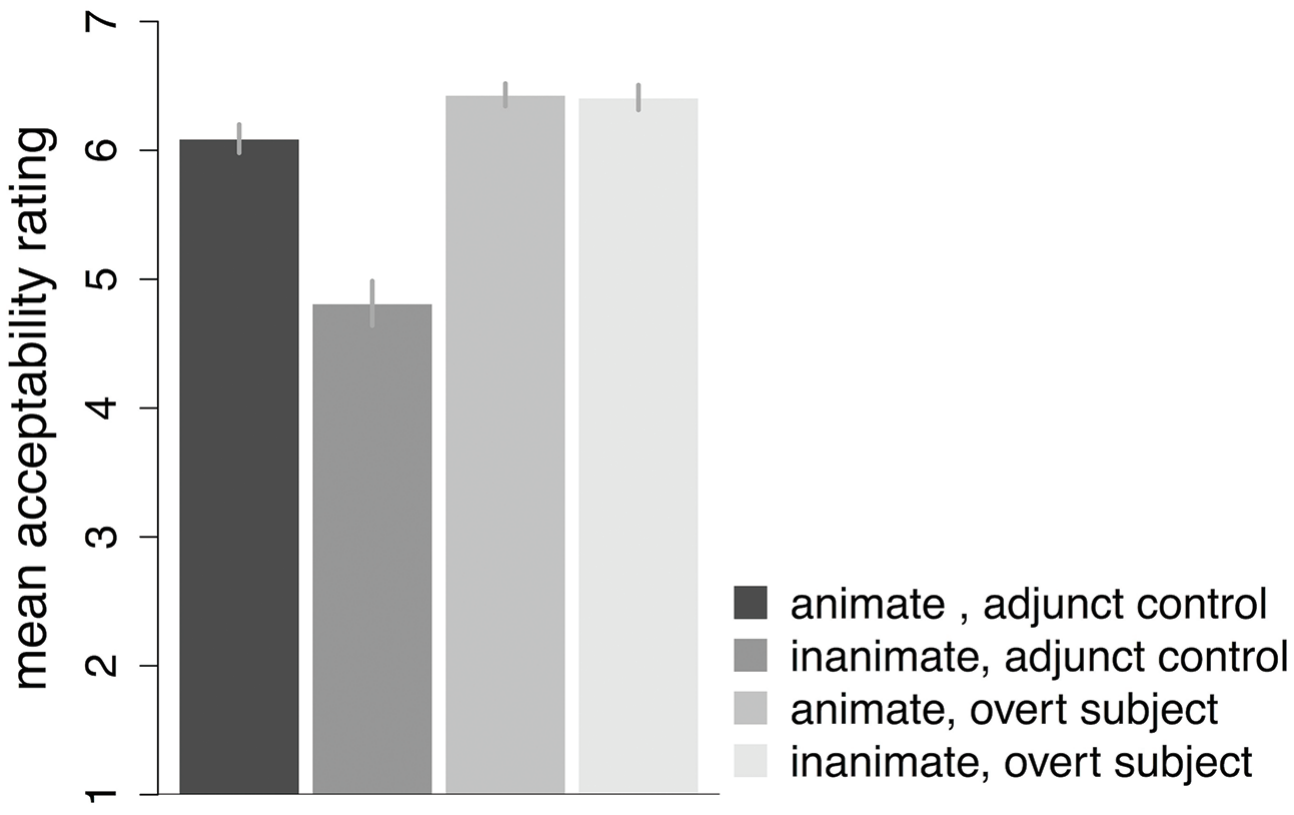
**Mean ratings and SE by participants for Experiment 1.** Values are on a 7-point Likert scale, with ‘7’ being most acceptable, and ‘1’ the least acceptable. Error bars represent SEM.

The results of Experiment 1 are presented in **Figure 1**. Adjunct control sentences with animate subjects were rated higher than those with inanimate subjects (means: 4.81 inanimate subject vs. 6.09 animate subject). By contrast, sentences with animate and inanimate overt subjects received similar ratings (means: 6.43 inanimate subject vs. 6.40 animate subject). The statistical analysis revealed a main effect of subject ANIMACY (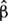 = -0.64, SE = 0.19, *t* = -3.40), a main effect of CONSTRUCTION (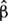 = -0.96, SE = 0.18, *t* = -5.08), and an interaction between subject ANIMACY and CONSTRUCTION (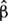 = -1.25, SE = 0.30, *t* = -4.08). The interaction was driven by the fact that animacy significantly modulated ratings in the adjunct control conditions (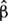 = -1.26, SE = 0.29, *t* = -4.32), but not in the overt subject conditions (*t* < 2).

### Discussion

Experiment 1 confirmed that adjunct control sentences are more acceptable with animate than with inanimate licensors. However, since sentences with inanimate licensors received relatively high ratings, we believe that the animacy constraint should be regarded as a weak constraint for adjunct control, or that it is a constraint that has a smaller impact on ratings because it does not block interpretability. Furthermore, the finding that the animacy manipulation did not impact ratings for sentences with an overt embedded subject implies that the animacy preference for adjunct control cannot simply reflect a general property of embedded clauses or lexical verb biases. Based on these findings, we conclude that comprehenders might use animacy as a cue to guide memory retrieval for null subject licensing, as has been reported for other linguistic dependencies, such as thematic binding (e.g., Van Dyke and Lewis, 2003; Van Dyke and McElree, 2006, 2011).

## Experiment 2

The goal of Experiment 2 was to test the hypothesis that all anaphoric dependencies resist facilitatory interference during real-time comprehension. We used self-paced reading to investigate whether retrieval for null subject licensing is susceptible to interference from animate distractors in structurally inappropriate locations. Under the hypothesis that all anaphoric dependencies are immune to facilitatory interference, retrieval for null subject licensing should avoid facilitatory interference from structurally inappropriate animate distractors. Alternatively, if this hypothesis is incorrect, then we might observe facilitatory interference, yielding a profile similar to subject–verb agreement.

### Participants

Thirty-two members of the University of Maryland community participated in Experiment 2. Participants were either compensated $10 or received credit in an introductory linguistics course. The self-paced reading task lasted approximately 40 min and was administered as part of a 1-hour session involving unrelated experiments.

### Materials

The experimental materials consisted of 48 item sets, each containing eight conditions. The experimental conditions consisted of a 2 × 2 × 2 factorial design, which crossed the factors DEPENDENCY, GRAMMATICALITY, and DISTRACTOR. An example item set is provided in **Table 1**. The first factor, DEPENDENCY, varied the dependency of interest: adjunct control vs. subject–verb agreement. Subject–verb agreement conditions were included to provide an experiment-internal measure of facilitatory interference effects. Within each dependency type, the sentences were maximally similar and differed only in the manipulations of GRAMMATICALITY and DISTRACTOR.

**Table 1:** Example set of experimental items for Experiment 2.

**Adjunct control conditions**
**Grammatical, distractor** The doctor that the researcher described meticulously was certified after debunking the urban myth himself in the new scientific journal.
**Grammatical, no distractor** The doctor that the report described meticulously was certified after debunking the urban myth himself in the new scientific journal. **Ungrammatical, distractor** The discovery that the researcher described meticulously was certified after debunking the urban myth himself in the new scientific journal. **Ungrammatical, no distractor** The discovery that the report described meticulously was certified after debunking the urban myth himself in the new scientific journal.
**Subject–verb agreement conditions**
**Grammatical, distractor** The doctor that the researcher described meticulously was certified after debunking the urban myth in the new scientific journal. **Grammatical, no distractor** The doctor that the reports described meticulously was certified after debunking the urban myth in the new scientific journal. **Ungrammatical, distractor** The doctor that the researchers described meticulously were certified after debunking the urban myth in the new scientific journal. **Ungrammatical, no distractor** The doctor that the report described meticulously were certified after debunking the urban myth in the new scientific journal.

All test items consisted of a passive main clause followed by an adjunct clause. Passive sentences were used because they naturally allow both animate and inanimate NPs in the main clause subject position, and provide a clear attachment site for the adjunct clause to the main clause VP, avoiding the possibility of an attachment ambiguity. In all conditions, the main clause subject was modified by an object relative clause that contained the distractor in subject position. The relative clause verb never overtly expressed agreement, and was always followed by an adverbial that signaled the end of the relative clause. The main clause verb phrase consisted of an auxiliary form of *be* (*was* or *were*) immediately followed by the main verb and an adjunct clause that consisted of a subordinator and gerundive verb.

In the adjunct control conditions, the adjunct clause contained an emphatic reflexive that was licensed by the subject of the adjunct clause, i.e., the null subject. This configuration provided two points to measure susceptibility to facilitatory interference in the adjunct control conditions. The earliest point to measure the impact of the distractor was the gerundive verb. The second point was the emphatic reflexive. Since the reflexive must access the properties of the adjunct clause subject, it was meant to provide a probe of the properties of the licensor retrieved for the anaphoric null subject. In the subject–verb agreement conditions, the earliest point to measure susceptibility to facilitatory interference was the main clause verb.

The factor GRAMMATICALITY was manipulated by varying the animacy of the main clause subject in the adjunct control conditions and the number of the agreeing verb in the subject–verb agreement conditions. In the grammatical adjunct control conditions, the main clause subject was animate and matched the animacy of the reflexive, which satisfied the animacy requirement of the adjunct control structures. In the ungrammatical conditions, the main clause subject did not satisfy the animacy requirement and mismatched the reflexive in animacy. In the grammatical subject–verb agreement conditions, the main clause subject and the agreeing verb were always singular, and thus matched in number. In the ungrammatical conditions, the agreeing verb was plural and mismatched the number of the main clause subject. Lastly, the factor DISTRACTOR was manipulated by varying the animacy of the distractor in the adjunct control conditions and the number of the distractor in the subject–verb agreement conditions. In order to avoid spurious effects due to lexical differences, the lexical content of the main clause was held constant across dependencies.

### Procedure

Sentences were presented on a desktop PC in a moving-window self-paced reading display using Linger (Doug Rohde). Sentences were initially masked by dashes, with white spaces and punctuation intact. Participants pushed the space bar to reveal each word. Presentation was non-cumulative, such that the previous word was replaced with a dash when the next word appeared. Each sentence was followed by a ‘yes/no’ comprehension question, and onscreen feedback was provided for incorrect answers. The order of presentation was randomized for each participant.

### Data Analysis

Only data from participants with at least 70% accuracy on the comprehension questions were used in the analysis. No participants were excluded due to poor accuracy. Reading times greater than 2500 ms were excluded from the analysis (following Hofmeister, 2011; Vasishth and Drenhaus, 2011). This trimming method affected less than 1% of the data. Reading times were then log-transformed to reduce non-normality. For the adjunct control conditions average reading times were compared between conditions in four regions of interest: the subordinator (v-1), the gerundive verb (v), the emphatic reflexive (refl), and the word immediately following the reflexive (refl + 1). For the subject–verb agreement conditions, average reading times were compared between conditions in two regions of interest: the agreeing verb (v) and the main verb (v + 1).

Reading time data were analyzed using linear mixed-effects models. Experimental fixed effects and their interaction were set up using orthogonal contrast coding, and items and participants were crossed as random effects (Baayen et al., 2008). To determine whether inclusion of random slopes was necessary, we compared an intercept-only model to a model with a fully specified random effects structure, which included random intercepts and slopes for all fixed effects and their interaction by items and by participants. A log-likelihood ratio test revealed that the maximal model did not provide a better fit to the data in the critical regions [subject–verb agreement: χ^2^_(18)_ = 4.39, *p* = 0.92; adjunct control: χ^2^_(18)_ = 9.98, *p* = 0.93]. Therefore, we adopted the intercept-only model, and for consistency, we applied the same model to all regions of interest.

### Results

#### Subject–Verb Agreement Conditions

**FIGURE 2:**
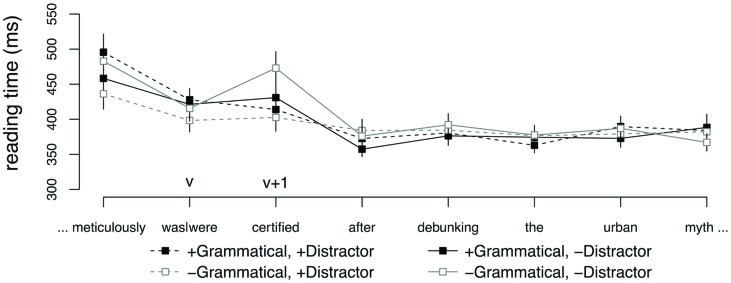
**Word-by-word reading times for subject–verb agreement conditions, Experiment 2.** Error bars indicate SEM.

**Figure 2** shows average reading times starting from the region preceding the agreeing verb to five regions beyond the main verb. No effects were observed at the critical verb (v). The word immediately following the critical verb (v + 1) showed a main effect of DISTRACTOR (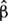 = 0.06, SE = 0.02, *t* = -2.22) and crucially, an interaction between GRAMMATICALITY and DISTRACTOR (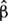 = -0.17, SE = 0.05, *t* = -2.96). This interaction was driven by a significant effect of DISTRACTOR in the ungrammatical conditions (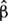 = -0.15, SE = 0.04, *t* = -3.56), reflecting faster reading times for sentences with a plural distractor, relative to sentences with no distractor. No such difference was observed in the grammatical conditions (*t* < 2).

#### Adjunct Control Conditions

**FIGURE 3:**
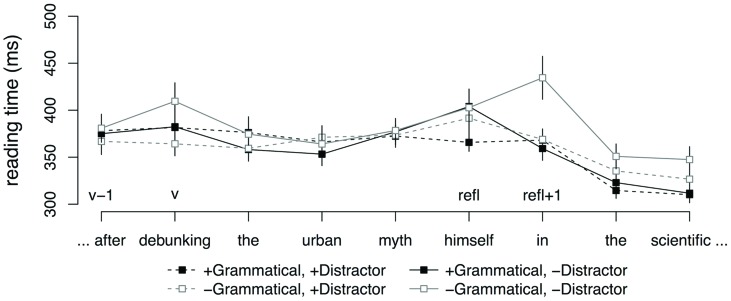
**Word-by-word reading times for adjunct control conditions, Experiment 2.** Error bars indicate SEM.

**Figure 3** shows average reading times starting from the subordinator to three regions following the reflexive. No effects were observed at the subordinator region (v-1). At the gerundive verb (v), there was an interaction between GRAMMATICALITY and DISTRACTOR (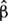 = -0.11, SE = 0.04, *t* = -2.48). This interaction was driven by a significant effect of DISTRACTOR in the ungrammatical conditions (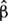 = -0.07, SE = 0.03, *t* = -2.03), reflecting faster reading times for sentences with an animate distractor relative to sentences with an inanimate distractor. No such difference was observed for the grammatical conditions (*t* < 2). No effects were observed at the reflexive (refl). The word immediately following the reflexive (refl + 1) showed a main effect of GRAMMATICALITY (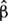 = -0.06, SE = 0.02, *t* = -3.02) and an interaction between GRAMMATICALITY and DISTRACTOR (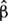 = -0.10, SE = 0.04, *t* = -2.23). The main effect of GRAMMATICALITY was due to slower reading times in the ungrammatical conditions relative to the grammatical conditions. The interaction was driven by a significant effect of DISTRACTOR in the ungrammatical conditions (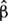 = -0.07, SE = 0.03, *t* = -2.02), reflecting faster reading times for sentences with an animate distractor relative to sentences with no distractor. No such difference was observed for the grammatical conditions (*t* < 2).

### Discussion

Experiment 2 tested the hypothesis that immunity to facilitatory interference is a general property of anaphoric dependencies. Our results provide evidence against this hypothesis, since they show that adjunct control dependencies, which involve an anaphoric relation between a null subject and its licensor, are susceptible to facilitatory interference, similarly to subject–verb agreement. Facilitatory interference was observed at two different points in the adjunct control sentences. The first was at the gerundive verb, which was the earliest point where sensitivity to the structurally inappropriate distractor could be detected. At this region, reading times for ungrammatical sentences were facilitated by the presence of a structurally inappropriate animate distractor, leading to an illusion of grammaticality. The second region was the reflexive, which served as an additional probe of the properties of the licensor that was retrieved for null subject licensing. Reading times at this region showed a similar profile to the gerundive verb with respect to facilitatory interference. Taken together, these findings suggest that the structurally inappropriate animate distractor was sometimes retrieved as the subject of the adjunct clause, which licensed the reflexive without detection of the animacy violation.

The finding that null subject licensing exhibits facilitatory interference effects is striking, given that such robust effects have rarely been observed for anaphora before. Previous studies have consistently failed to find evidence of facilitatory interference in the comprehension of anaphoric dependencies, such as those involving direct object reflexives. In contrast, we found that null subject licensing shows an interference profile that is qualitatively similar to that observed for subject–verb agreement dependencies, which show strong interference effects.

The findings from Experiment 2 showed facilitatory interference for null subjects in adjunct control structures. However, our interpretation of the interference profile at the emphatic reflexive is based on the assumption that the reflexive was a faithful reflection of what was retrieved as the subject of the adjunct clause at the gerundive. This assumption is based on previous findings that reflexives generally only search for a licensor within the domain of their local clause (e.g., Sturt, 2003; Dillon et al., 2013). However, an alternative explanation of our results is that the interference effects observed at the gerundive and reflexive reflect independent effects, and that the profile observed at the reflexive is not predicated on the outcome of null subject licensing at the gerundive. For instance, the reflexive may not have tracked the interpretation of the subject of the adjunct clause but rather linked directly to one of the NPs in the higher clause (e.g., *the doctor*, *the report*). Since little is known about the processing of emphatic reflexives, it is possible that, unlike direct object reflexives, emphatic reflexives may trigger an error-prone retrieval that is not constrained to the domain of the adjunct clause, thus giving rise to an interference effect that is independent of the outcome of null subject licensing. We tested this possibility in Experiment 3.

## Experiment 3

Experiment 3 tested the assumption that the reflexive in Experiment 2 tracked the interpretation of the subject of the adjunct clause, rather than linking directly to one of the NPs in the higher clause. We reasoned that if the reflexive accurately reflected the interference effect observed for null subject licensing, then eliminating interference for null subject licensing should also eliminate interference at the reflexive. To achieve this, we held constant the animacy of the target NP in the main clause and distractor NP in the relative clause, and manipulated their gender match with the reflexive instead, as shown in (6).

(6)  The (harpist|drummer) that the (diva|guitarist) liked very much was congratulated after playing the beautiful song **herself** at the brand new recording studio.

As described earlier, reflexives require gender agreement with a licensor, but null subjects do not. Thus, the gender manipulation in (6) should not generate any interference effects at the gerundive in the adjunct clause, as only the correct licensor (*harpist*/*drummer*) should be retrieved for null subject licensing. Further, if the reflexive is a faithful reflection of what was retrieved for null subject licensing, then the reflexive should only be sensitive to the gender match of the structurally appropriate licensor (*harpist* vs. *drummer*), and thus pattern with the gerundive in the absence of interference effects. If, on the other hand, the reflexive links directly to either of the NPs in the higher clause, then different profiles might be obtained for null subject and reflexive licensing. In particular, although we do not expect interference at the gerundive, we might observe an interference effect at the reflexive when there is a structurally inappropriate but gender matching distractor in the relative clause (e.g., *diva*).

### Participants

Thirty-two members of the University of Maryland community participated in Experiment 3. Participants were either compensated $10 or received credit in an introductory linguistics course. The task lasted approximately 40 min and was administered as part of a 1-hour session involving unrelated experiments.

### Materials

The design of Experiment 3 was the same as Experiment 2, except that the animacy of the target and distractor NPs was held constant, and their gender match to the reflexive was manipulated. The experimental materials consisted of 48 item sets, each containing eight conditions. The experimental conditions consisted of a 2 × 2 × 2 factorial design, which crossed the factors DEPENDENCY, GRAMMATICALITY, and DISTRACTOR. An example item set is provided in **Table 2**. As in Experiment 2, the target NP appeared as the subject of the main clause, and was modified by an object relative clause that contained the distractor in subject position. The factor DEPENDENCY compared adjunct control conditions with subject–verb agreement conditions. The factor GRAMMATICALITY was manipulated by varying the stereotypical gender of the main clause subject in the adjunct control conditions and the number of the agreeing verb in the subject–verb agreement conditions. In the grammatical adjunct control conditions, the main clause subject was animate and matched the gender of the reflexive. In the ungrammatical conditions, the main clause subject was animate, but mismatched the gender of the reflexive. In the grammatical subject–verb agreement conditions, the agreeing verb was always singular and matched the number of the main clause subject. In the ungrammatical conditions, the agreeing verb was plural and mismatched the number of the main clause subject. The factor DISCTRACTOR was manipulated by varying the stereotyped gender of the distractor for adjunct control conditions and the number of the distractor for subject–verb agreement conditions. As in Experiment 2, the lexical content of the main clause was held constant across dependency types to avoid spurious effects due to lexical differences.

**Table 2:** Example set of experimental items for Experiment 3.

**Adjunct control conditions**
**Grammatical, distractor** The harpist that the diva liked very much was congratulated after playing the beautiful song herself at the brand new recording studio.
**Grammatical, no distractor** The harpist that the guitarist liked very much was congratulated after playing the beautiful song herself at the brand new recording studio. **Ungrammatical, distractor** The drummer that the diva liked very much was congratulated after playing the beautiful song herself at the brand new recording studio. **Ungrammatical, no distractor** The drummer that the guitarist liked very much was congratulated after playing the beautiful song herself at the brand new recording studio.
**Subject–verb agreement conditions**
**Grammatical, distractor** The harpist that the diva liked very much was congratulated after playing the beautiful song at the brand new recording studio. **Grammatical, no distractor** The harpist that the divas liked very much was congratulated after playing the beautiful song at the brand new recording studio. **Ungrammatical, distractor** The harpist that the divas liked very much were congratulated after playing the beautiful song at the brand new recording studio. **Ungrammatical, no distractor** The harpist that the diva liked very much were congratulated after playing the beautiful song at the brand new recording studio.

### Procedure

The same self-paced reading procedure was used as in Experiment 2.

### Data Analysis

The statistical analysis followed the same steps as in Experiment 2. Four participants were excluded from the analysis due to accuracy below 70% in the comprehension questions. Data trimming affected less than 1% of the data. Model comparisons revealed that a maximally specified random effects structure did not provide a better fit to the data in the critical regions than an intercept-only model [subject–verb agreement: χ^2^_(18)_ = 11.16, *p* = 0.88; adjunct control: χ^2^_(18)_ = 14.53, *p* = 0.69]. Therefore, we adopted the intercept-only model.

### Results

#### Subject–Verb Agreement Conditions

**FIGURE 4:**
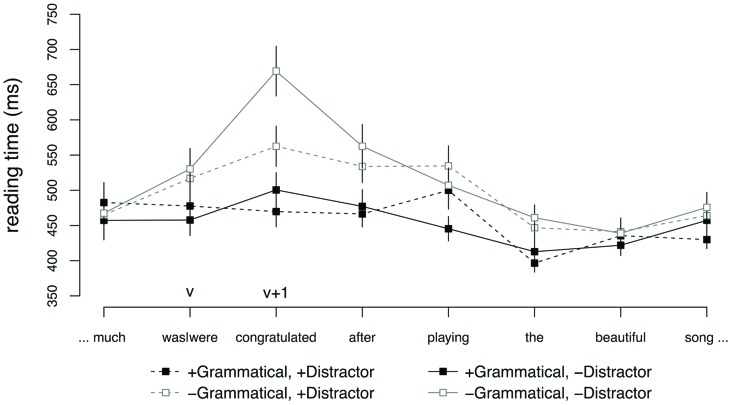
**Word-by-word reading times for subject–verb agreement conditions, Experiment 3.** Error bars indicate SEM.

**Figure 4** shows average reading times starting from the region preceding the agreeing verb to five regions following the main verb. No effects were observed at the critical verb (v). The word immediately following the critical verb (v + 1) showed a main effect of GRAMMATICALITY (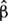 = 0.18, SE = 0.03, *t* = -5.06) and, crucially, an interaction between GRAMMATICALITY and DISTRACTOR (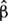 = -0.21, SE = 0.07, *t* = -2.98). This interaction was driven by a significant effect of DISTRACTOR in the ungrammatical conditions (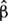 = -0.16, SE = 0.05, *t* = -2.97), reflecting faster reading times for sentences with a plural distractor relative to sentences with no distractor. No such difference was observed for the grammatical sentences (*t* < 2).

#### Adjunct Control Conditions

**FIGURE 5:**
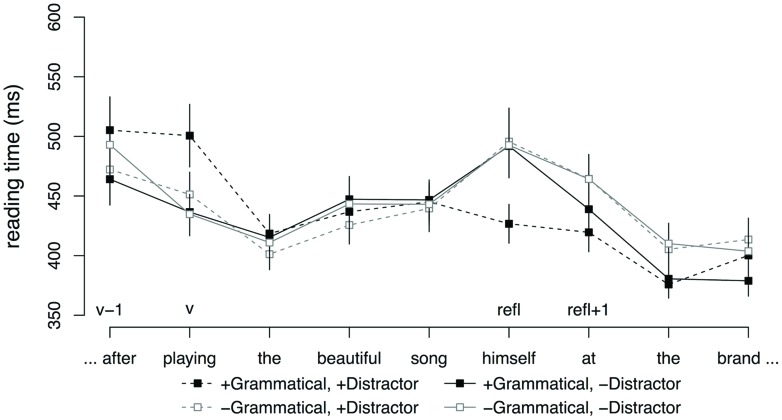
**Word-by-word reading times for adjunct control conditions, Experiment 3.** Error bars indicate SEM.

**Figure 5** shows average reading times starting from the subordinator to three regions following the reflexive. No effects were observed at the subordinator (v-1). At the gerundive verb (v), there was a main effect of distractor (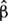 = -0.06, SE = 0.02, *t* = -2.31). Pairwise comparisons revealed that this effect was due to a slowdown for grammatical conditions with an animate distractor (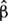 = 0.10, SE = 0.04, *t* = 2.33). No effect was observed in the ungrammatical conditions (*t* < 2). At the reflexive (refl), the grammatical condition with an animate distractor showed faster reaction times (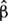 = -0.09, SE = 0.03, *t* = -2.41). No other effects were observed at the reflexive (all *ts* < 2). The word immediately following the reflexive (refl + 1) showed a main effect of GRAMMATICALITY (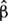 = -0.05, SE = 0.02, *t* = -2.09), reflecting a slowdown for ungrammatical conditions relative to grammatical conditions. Crucially, and in contrast with Experiment 2, there was no effect of facilitatory interference at the word following the reflexive and no interaction was observed between GRAMMATICALITY and DISTRACTOR.

### Discussion

Experiment 3 tested the assumption that the reflexive in the adjunct control constructions in Experiment 2 was a faithful reflection of what was previously retrieved as the subject of the adjunct clause. We reasoned that if the interference effect seen at the reflexive in Experiment 2 reflected the interference effect observed for subjects at the gerundive verb, then eliminating interference at the gerundive verb should also eliminate interference at the reflexive. This outcome is not obvious, since different features are required to match to license the gerundive (animacy) and the reflexive (animacy, number, gender). As predicted, eliminating interference for null subject licensing also eliminated interference at the reflexive. These results provide preliminary evidence that the reflexive tracked the interpretation of the subject of the adjunct clause, rather than directly linking to one of the NPs in the higher clause. We discuss this further in the General Discussion.

Experiment 3 also revealed a main effect of distractor at the gerundive verb and the reflexive regions. Specifically, the presence of multiple gender matched licensors in the grammatical conditions (e.g., *The harpist that the diva… after playing… herself*) increased reading times at the gerundive verb, and later facilitated reading times in the same conditions at the reflexive. These effects were unexpected, and we believe that the effect of distractor at the gerundive might reflect a “fan” effect (Anderson, 1974; Anderson and Reder, 1999), which can arise in grammatical contexts when multiple items match the retrieval cues (Badecker and Straub, 2002; Autry and Levine, 2014; but cf. Chow et al., 2014).

In contrast with facilitatory interference effects at retrieval, fan effects have been argued to reflect interference at the encoding stage (Dillon, 2011). For example, encountering multiple items that overlap in morphological features can degrade the quality of memory representations for those items due to feature-overwriting (Nairne, 1988, 1990). Thus, the reading time slowdown at the gerundive for grammatical sentences with multiple match items may reflect impeded access to a degraded memory representation of the target at the point of retrieval for null subject licensing. Crucially, this effect does not entail that the structurally inappropriate licensor was retrieved during null subject licensing (see Dillon, 2011 for discussion). By contrast, the facilitation in the same conditions later at the reflexive could reflect a gender familiarity effect. After reading the gerundive verb, comprehenders might have been fairly confident that a gender matching item (*harpist* and *diva*) was present in the sentence, leading to facilitated processing (i.e., faster reading times) at the reflexive.

In sum, the critical finding from Experiment 3 is the absence of facilitatory interference effects in the ungrammatical conditions at the reflexive. These findings provide preliminary evidence that the reflexive in the adjunct control constructions from Experiment 2 tracked the interpretation of the subject of the same clause, rather than linking directly to one of the NPs in the higher clause. However, further research is necessary to better understand the source of the facilitation effect in the grammatical conditions.

## General Discussion

### Summary of Findings

The present study addressed one part of the puzzle of why reflexives and subject–verb agreement show contrasting profiles with respect to facilitatory interference effects. We tested the hypothesis that all anaphoric dependencies resist facilitatory interference from structurally inappropriate items during real-time comprehension. Specifically, we used an animacy manipulation to examine whether adjunct control dependencies, which involve an interpreted anaphoric relation between a null subject and its licensor, behave like reflexives in that they are immune to facilitatory interference effects.

**FIGURE 6:**
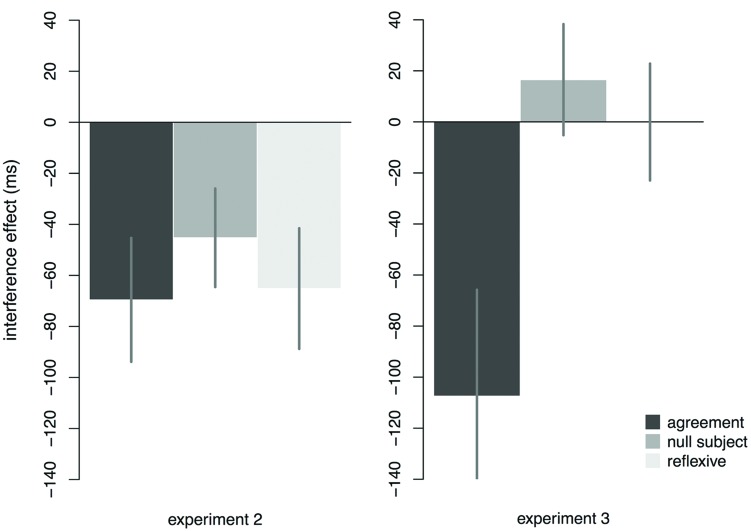
**Comparison of interference effects observed for subject-verb agreement (region: v + 1), null subject licensing (region: v), and reflexive licensing (region: refl + 1) in Experiments 2 and 3.** Error bars indicate SEM. Interference effects (in ms) were estimated as the difference between the means of the two ungrammatical conditions.

In Experiment 1, we confirmed that null subject licensing in adjunct control structures obeys an animacy requirement, which we then used as a probe for interference effects in Experiment 2. In Experiment 2, we directly compared the reading time profiles of null subject licensing and subject–verb agreement dependencies. Our results revealed qualitatively similar profiles with respect to facilitatory interference, as illustrated in **Figure 6**. Specifically, we found reliable interference effects for null subject licensing at two points: at the gerundive verb and later, at a reflexive within the same clause, which served as an additional probe of what was retrieved as the subject of the gerundive verb.

The results from Experiment 2 challenge the hypothesis that all anaphoric dependencies resist facilitatory interference. Specifically, our results suggest that anaphors do not behave homogenously with respect to facilitatory interference, since null subject anaphors show interference, whereas reflexive anaphors typically do not. Thus, we believe that any account that claims that interference effects are linked to specific types of grammatical dependencies (e.g., anaphora vs. agreement) is unlikely to be successful. Furthermore, the results of Experiment 2 challenge the hypothesis that the contrast between reflexives and agreement seen in previous studies reflects differences based on their interpretive status. According to this hypothesis, anaphoric dependencies might engage a more conservative retrieval strategy to avoid misinterpretation and interference from structurally inappropriate items. Our results provide evidence against this hypothesis by showing that interpreted anaphoric dependencies involving null subjects are susceptible to facilitatory interference.

In Experiment 3, we tested the assumption that the reflexive in Experiment 2 tracked the interpretation of the subject of the same clause, rather than linking directly to one of the NPs in the higher clause. We tested a configuration that did not yield interference for null subject licensing, and found that the corresponding interference effect at the reflexive also disappeared, as shown in **Figure 6**. These results suggest that our assumption was justified.

However, there is an alternative explanation for the contrasting profiles at the reflexive between Experiments 2 and 3. Whereas in Experiment 2 the reflexive mismatched its licensor in both animacy and gender, in Experiment 3, the reflexive only mismatched its licensor in gender. This raises the possibility that the contrasting profiles at the reflexive between Experiments 2 and 3 may not reflect differences based on the outcome of null subject licensing. Rather, the contrast may have been caused by differences based on the degree of match between the reflexive and the candidate licensors (i.e., 2-feature mismatch in Experiment 2, but 1-feature mismatch in Experiment 3). Specifically, it is possible that the feature matching distractor was able to outcompete the target when the target mismatched the reflexive in 2 features, but not when it mismatched the reflexive in only 1 feature. This difference could lead to interference in a 2-feature mismatch context (Experiment 2), but not in a 1-feature mismatch context (Experiment 3). We discuss this possibility further below.

### Variability within Anaphora

The present study revealed that null subjects are susceptible to facilitatory interference in comprehension. These findings contrast with previous findings for reflexives, which typically resist interference. This raises the question of why anaphoric dependencies should behave differently at retrieval. We believe that there are two possibilities for why we should see variability within anaphora with respect to facilitatory interference.

First, previous studies on anaphora have failed to find evidence of facilitatory interference with designs that manipulated the gender or number match between the anaphor and its licensor. In contrast, we found evidence of facilitatory interference when we manipulated animacy. It is possible that the interference effects in our study reflect an inherent primacy of animacy information in anaphoric licensing. This could arise if animacy is a more reliable cue to the target subject in comprehension. For example, whereas a subject in a licensor position is typically animate, its gender and number may be more variable, leading comprehenders to prioritize animacy information at retrieval to access the target subject. This hypothesis aligns with recent findings on the psychology of memory, which suggest that animacy information is one of the most important dimensions in controlling memory retention (Nairne et al., 2013; Van Arsdall et al., 2013).

A second possibility is that the variability across studies could reflect the degree of feature match between the anaphor and its licensor (i.e., probe-to-target similarity). This possibility was raised earlier in our discussion of the contrasting profiles at the reflexive between Experiments 2 and 3. Specifically, we observed facilitatory interference at the reflexive when the target licensor mismatched the reflexive in two features (i.e., both gender and animacy), but failed to find evidence of interference when the target licensor mismatched the reflexive in only one feature (i.e., gender). These findings suggest that retrieval for reflexive processing might only be susceptible to facilitatory interference in configurations where the target mismatches the reflexive in more than one feature^6^.

Our findings do not distinguish between the two possibilities discussed above, but they suggest some further directions. One avenue for future research would be to focus on the processing of direct object reflexives and to compare contexts in which the reflexive-antecedent dependency involves 1 vs. 2 feature mismatches. This test could help determine the source of the interference effects at the reflexive in Experiment 2 (see Parker and Phillips, 2014). Another avenue for future research would be to test the hypothesis that interference in anaphora is due to the privileged use of animacy information in retrieval. To achieve this, one could test languages where animacy and gender are not conflated, like Spanish or Polish. In these languages, gender is a syntactic property that is distinct from stereotypical or conceptual gender, such that a mismatch in animacy between an anaphor and its licensor does not entail a gender mismatch, like in English. Testing the impact of animacy independently of gender in these languages could help determine whether there is an inherent primacy of animacy in retrieval for anaphor processing.

## Conclusion

This study explored the hypothesis that all anaphoric dependencies resist facilitatory interference during real-time comprehension. Our results challenged this hypothesis by showing that anaphoric dependencies do not behave homogenously with respect to facilitatory interference effects. Specifically, we found that adjunct control dependencies, which involve an anaphoric relation between a null subject and a licensor, are susceptible to facilitatory interference. In discussion, we explored several options for why anaphoric dependencies should vary with respect to facilitatory interference. We argued that variability within anaphora could reflect either an inherent primacy of specific content cues like animacy in retrieval processes, or the differential degree of match between the potential licensors and retrieval probe.

## Conflict of Interest Statement

The authors declare that the research was conducted in the absence of any commercial or financial relationships that could be construed as a potential conflict of interest.

## Footnotes

1 Facilitatory interference cannot be simply a case of proximity concord (Quirk et al., 1985) or local coherence (Tabor et al., 2004), as the effect is also observed when the plural distractor does not intervene between the verb and true subject (cf. Wagers et al., 2009). The effect is also not merely due to dialectal variation, as speakers agree on the unacceptability of sentences like (1b) when they have ample time to make their judgment (see Dillon et al., 2013).

2 More specifically, the account proposed by Wagers et al. (2009) predicts a bimodal response from the mixture of two distributions: either retrieval recovers the structurally inappropriate distractor, facilitating reading times and triggering an illusion, or the structurally appropriate target, slowing down processing. As a result, the average reading times across trials are reduced for ungrammatical sentences with a feature matching distractor relative to ungrammatical sentences where no matching distractor is present.

3 Our discussion does not rely on whether the missing subject is an empty category, e.g., PRO. See Hornstein (2003) for a discussion of the debate over how to formally represent control clause subjects.

4 https://www.mturk.com.

5 http://spellout.net/ibexfarm

6 A third possibility suggested by one of the reviewers is that retrieval involving animacy may be privileged over retrieval involving other features like gender or number, not because of the information-theoretic status of the antecedent, but because of the linguistic representation of feature hierarchies. Feature geometric approaches to agreement, e.g., Harley and Ritter (2002), propose that some features are represented as structurally higher than others, and we might expect that there are linguistic constraints on the order of feature access during retrieval.
