# Supplementary material for: Interference in the processing of adjunct control
Source: Front Psychol. 2015 Sep 8;6:1346. doi: 10.3389/fpsyg.2015.01346 (PMC4561755; doi:10.3389/fpsyg.2015.01346)
Supplement: Supplementary file 1 [file Data_Sheet_1.PDF]

Experimental Items
Parker, Lago, & Phillips
Interference in the processing of adjunct control

Items from Experiment 1

a Adjunct control, animate licensor
b Adjunct control, inanimate licensor
c Full embedded clause, overt animate subject
d Full embedded clause, overt inanimate subject

1 a The senator was criticized after promoting the controversial labor union.
1 b The law was criticized after promoting the controversial labor union.
1 c The public was displeased that the senator promoted the controversial labor union.
1 d The public was displeased that the law promoted the controversial labor union.
2 a The guitarist was praised after dispelling the horrible rumors.
2 b The magazine was praised after dispelling the horrible rumors.
2 c The drummer was happy that the guitarist dispelled the rumors.
2 d The drummer was happy that the magazine dispelled the rumors.
3 a The nurse was deplored after revealing the dark secret.
3 b The tabloid was deplored after revealing the dark secret.
3 c The actress was upset that the nurse revealed the dark secret.
3 d The actress was upset that the tabloid revealed the dark secret.
4 a The director was appreciated after promoting the new movie.
4 b The article was appreciated after promoting the new movie.
4 c The actor was pleased that the director promoted the new movie.
4 d The actor was pleased that the article promoted the new movie.
5 a The butcher was criticized after failing the health inspection.
5 b The meat was criticized after failing the health inspection.
5 c The restaurant owner was very mad that the butcher failed the health inspection.
5 d The restaurant owner was very mad that the meat failed the health inspection.
6 a The professor was noted after solving the complex problem.
6 b The book was noted after solving the complex problem.
6 c The students were impressed that the professor solved the problem.
6 d The students were impressed that the book solved the problem.
7 a The secretary was promoted after eliminating several complicated tasks.
7 b The software was promoted after eliminating several complicated tasks.
7 c The manager was impressed that the secretary eliminated several complicated tasks.
7 d The manager was impressed that the software eliminated several complicated tasks.
8 a The housewife was disliked after causing several major problems.
8 b The law was disliked after causing several major problems.
8 c The lawyer was furious that the housewife caused several major problems.
8 d The lawyer was furious that the law caused several major problems.
9 a The politician was trusted after solving the health crisis.
9 b The plan was trusted after solving the health crisis.
9 c The governor was relieved that the politician solved the health crisis.
9 d The governor was relieved that the plan solved the health crisis.
10 a The supermodel was applauded after showcasing the fabulous wardrobe.
10 b The play was applauded after showcasing the fabulous wardrobe.
10 c The fashion designer was excited that the supermodel showcased the fabulous wardrobe.

10 d The fashion designer was excited that the play showcased the fabulous wardrobe.
11 a The statesman was appreciated after resolving the financial problem.
11 b The law was appreciated after resolving the financial problem.
11 c The senator was glad that the statesman resolved the financial problem.
11 d The senator was glad that the law resolved the financial problem.
12 a The lawyer was dismissed after verifying the disputed testimony.
12 b The evidence was dismissed after verifying the disputed testimony.
12 c The judge was convinced that the lawyer verified the disputed testimony.
12 d The witness was convinced that the evidence verified the disputed testimony.
13 a The doctor was commended after disproving the controversial theory.
13 b The experiment was commended after disproving the controversial theory.
13 c The journalist was impressed that the doctor disproved the controversial theory.
13 d The journalist was impressed that the experiment disproved the controversial theory.
14 a The congressman was denounced after introducing several unnecessary restrictions.
14 b The policy was denounced after introducing several unnecessary restrictions.
14 c The community was displeased that the congressman introduced several unnecessary restrictions.
14 d The community was displeased that the policy introduced several unnecessary restrictions.
15 a The actor was nominated after portraying several important roles.
15 b The film was nominated after portraying several important roles.
15 c The critics were impressed that the actor portrayed several important roles.
15 d The critics were impressed that the film portrayed several important roles.
16 a The congressman was opposed before authoring the judicial appeal.
16 b The legislation was opposed before authorizing the judicial appeal.
16 c The mayor was furious that the congressman authorized the judicial appeal.
16 d The mayor was furious that the legislation authorized the judicial appeal.
17 a The representative was opposed after eliminating the after-school program.
17 b The contract was opposed after eliminating the after-school program.
17 c The high school principal was upset that the representative eliminated the after-school program.
17 d The high school principal was upset that the contract eliminated the after-school program.
18 a The architect was certified before improving the preliminary design.
18 b The blueprint was certified before improving the preliminary design.
18 c The engineer was pleased that the architect improved the preliminary design.
18 d The engineer was pleased that the blueprint improved the preliminary design.
19 a The activist was commended before starting the political movement.
19 b The manifesto was commended before starting the political movement.
19 c The historian was surprised that the protester started the political movement.
19 d The historian was surprised that the manifesto started the political movement.
20 a The schoolgirl was appreciated after completing many important tasks.
20 b The instrument was appreciated after completing many important tasks.
20 c The teacher was impressed that the schoolgirl completed many important tasks.
20 d The teacher was impressed that the instrument completed many important tasks.
21 a The officer was reviewed after revealing the crucial evidence.
21 b The letter was reviewed after revealing the crucial evidence.
21 c The criminal was displeased that the officer revealed the crucial evidence.
21 d The criminal was displeased that the letter revealed the crucial evidence.
22 a The janitor was certified before eradicating the filthy rats.
22 b The chemical was certified before eradicating the filthy rats.
22 c The landlord was reassured that the janitor eradicated the filthy rats.
22 d The landlord was reassured that the chemical eradicated the filthy rats.
23 a The newsman was ignored after dispelling the horrible rumor.

23 b The statement was ignored after dispelling the horrible rumor.
23 c The editor was relieved that the newsman dispelled the rumor.
23 d The editor was relieved that the statement dispelled the rumor.
24 a The engineer was approved before authorizing the large purchase.
24 b The document was approved before authorizing the large purchase.
24 c The manager was reassured that the engineer authorized the large purchase.
24 d The manager was reassured that the document authorized the large purchase.

Items from Experiment 2

a Adjunct control: Grammatical, distractor
b Adjunct control: Grammatical, no distractor
c Adjunct control: Ungrammatical, distractor
d Adjunct control: Ungrammatical, no distractor
e Subject-verb agreement: Grammatical, distractor
f Subject-verb agreement: Grammatical, no distractor
g Subject-verb agreement: Ungrammatical, distractor
h Subject-verb agreement: Ungrammatical, no distractor

1 a The senator that the journalist evaluated extensively was criticized for promoting the controversial labor union himself without any support from the executives.
1 b The senator that the magazine evaluated extensively was criticized for promoting the controversial labor union himself without any support from the executives.
1 c The law that the journalist evaluated extensively was criticized for promoting the controversial labor union himself without any support from the executives.
1 d The law that the magazine evaluated extensively was criticized for promoting the controversial labor union himself without any support from the executives.
1 e The senator that the journalist evaluated extensively was criticized for promoting the controversial labor union without any support from the executives.
1 f The senator that the magazines evaluated extensively was criticized for promoting the controversial labor union without any support from the executives.
1 g The law that the journalists evaluated extensively were criticized for promoting the controversial labor union without any support from the executives.
1 h The law that the magazine evaluated extensively were criticized for promoting the controversial labor union without any support from the executives.
2 a The guitarist that the publicist promoted widely was praised for dispelling the horrible rumors himself at the party for the executives.
2 b The guitarist that the magazine promoted widely was praised for dispelling the horrible rumors himself at the party for the executives.
2 c The album that the publicist promoted widely was praised for dispelling the horrible rumors himself at the party for the executives.
2 d The album that the magazine promoted widely was praised for dispelling the horrible rumors himself at the party for the executives.
2 e The guitarist that the publicist promoted widely was praised for dispelling the horrible rumors at the party for the executives.
2 f The guitarist that the magazines promoted widely was praised for dispelling the horrible rumors at the party for the executives.

2 g The album that the publicists promoted widely were praised for dispelling the horrible rumors at the party for the executives.

2 h The album that the magazine promoted widely were praised for dispelling the horrible rumors at the party for the executives.

3 a The beautician that the stylist bolstered proudly was endorsed after establishing the fashion trend herself without the use of expensive fabric.

3 b The beautician that the catalog bolstered proudly was endorsed after establishing the fashion trend herself without the use of expensive fabric.

3 c The company that the stylist bolstered proudly was endorsed after establishing the fashion trend herself without the use of expensive fabric.

3 d The company that the catalog bolstered proudly was endorsed after establishing the fashion trend herself without the use of expensive fabric.

3 e The beautician that the stylist bolstered proudly was endorsed after establishing the fashion trend without the use of expensive fabric.

3 f The beautician that the catalogs bolstered proudly was endorsed after establishing the fashion trend without the use of expensive fabric.

3 g The company that the stylists bolstered proudly were endorsed after establishing the fashion trend without the use of expensive fabric.

3 h The company that the catalog bolstered proudly were endorsed after establishing the fashion trend without the use of expensive fabric.

4 a The nurse that the secretary criticized harshly was deplored after revealing the dark secret herself at the expense of the doctor.

4 b The nurse that the tabloid criticized harshly was deplored after revealing the dark secret herself at the expense of the doctor.

4 c The affair that the secretary criticized harshly was deplored after revealing the dark secret herself at the expense of the doctor.

4 d The affair that the tabloid criticized harshly was deplored after revealing the dark secret herself at the expense of the doctor.

4 e The nurse that the secretary criticized harshly was deplored after revealing the dark secret at the expense of the doctor.

4 f The nurse that the tabloids criticized harshly was deplored after revealing the dark secret at the expense of the doctor.

4 g The affair that the secretaries criticized harshly were deplored after revealing the dark secret at the expense of the doctor.

4 h The affair that the tabloid criticized harshly were deplored after revealing the dark secret at the expense of the doctor.

5 a The director that the columnist reviewed critically was appreciated for promoting the new movie himself at the fancy theater across town.

5 b The director that the article reviewed critically was appreciated for promoting the new movie himself at the fancy theater across town.

5 c The preview that the columnist reviewed critically was appreciated for promoting the new movie himself at the fancy theater across town.

5 d The preview that the article reviewed critically was appreciated for promoting the new movie himself at the fancy theater across town.

5 e The director that the columnist reviewed critically was appreciated for promoting the new movie at the fancy theater across town.

5 f The director that the articles reviewed critically was appreciated for promoting the new movie at the fancy theater across town.

5 g The preview that the columnists reviewed critically were appreciated for promoting the new movie at the fancy theater across town.

5 h The preview that the article reviewed critically were appreciated for promoting the new movie at the fancy theater across town.

6 a The butcher that the cook recommended highly was criticized after failing the health inspection himself for the third time this year.

6 b The butcher that the restaurant recommended highly was criticized after failing the health inspection himself for the third time this year.

6 c The meat that the cook recommended highly was criticized after failing the health inspection himself for the third time this year.

6 d The meat that the restaurant recommended highly was criticized after failing the health inspection himself for the third time this year.

6 e The butcher that the cook recommended highly was criticized after failing the health inspection for the third time this year.

6 f The butcher that the restaurants recommended highly was criticized after failing the health inspection for the third time this year.

6 g The meat that the cooks recommended highly were criticized after failing the health inspection for the third time this year.

6 h The meat that the restaurant recommended highly were criticized after failing the health inspection for the third time this year.

7 a The chef that the waiter praised highly was acclaimed for setting the new standard himself at the very expensive restaurant uptown.

7 b The chef that the menu praised highly was acclaimed for setting the new standard himself at the very expensive restaurant uptown.

7 c The meal that the waiter praised highly was acclaimed for setting the new standard himself at the very expensive restaurant uptown.

7 d The meal that the menu praised highly was acclaimed for setting the new standard himself at the very expensive restaurant uptown.

7 e The chef that the waiter praised highly was acclaimed for setting the new standard at the very expensive restaurant uptown.

7 f The chef that the menus praised highly was acclaimed for setting the new standard at the very expensive restaurant uptown.

7 g The meal that the waiters praised highly were acclaimed for setting the new standard at the very expensive restaurant uptown.

7 h The meal that the menu praised highly were acclaimed for setting the new standard at the very expensive restaurant uptown.

8 a The professor that the researcher criticized harshly was noted for solving the complex problem himself without the use of complicated algorithms.

8 b The professor that the report criticized harshly was noted for solving the complex problem himself without the use of complicated algorithms.

8 c The book that the researcher criticized harshly was noted for solving the complex problem himself without the use of complicated algorithms.

8 d The book that the report criticized harshly was noted for solving the complex problem himself without the use of complicated algorithms.

8 e The professor that the researcher criticized harshly was noted for solving the complex problem without the use of complicated algorithms.

8 f The professor that the reports criticized harshly was noted for solving the complex problem without the use of complicated algorithms.

8 g The book that the researchers criticized harshly were noted for solving the complex problem without the use of complicated algorithms.

8 h The book that the report criticized harshly were noted for solving the complex problem without the use of complicated algorithms.

9 a The technician that the professor supported substantially was questioned after testing the controversial theory himself in the laboratory at the university.

9 b The technician that the university supported substantially was questioned after testing the controversial theory himself in the laboratory at the university.

9 c The research that the professor supported substantially was questioned after testing the controversial theory himself in the laboratory at the university.

9 d The research that the university supported substantially was questioned after testing the controversial theory himself in the laboratory at the university.

9 e The technician that the professor supported substantially was questioned after testing the controversial theory in the laboratory at the university.

9 f The technician that the universities supported substantially was questioned after testing the controversial theory in the laboratory at the university.

9 g The research that the professors supported substantially were questioned after testing the controversial theory in the laboratory at the university.

9 h The research that the university supported substantially were questioned after testing the controversial theory in the laboratory at the university.

10 a The secretary that the accountant mentioned briefly was promoted for eliminating several complicated tasks herself at the workshop for business management.

10 b The secretary that the grant mentioned briefly was promoted for eliminating several complicated tasks herself at the workshop for business management.

10 c The software that the accountant mentioned briefly was promoted for eliminating several complicated tasks herself at the workshop for business management.

10 d The software that the grant mentioned briefly was promoted for eliminating several complicated tasks herself at the workshop for business management.

10 e The secretary that the accountant mentioned briefly was promoted for eliminating several complicated tasks at the workshop for business management.

10 f The secretary that the grants mentioned briefly was promoted for eliminating several complicated tasks at the workshop for business management.

10 g The software that the accountants mentioned briefly were promoted for eliminating several complicated tasks at the workshop for business management.

10 h The software that the grant mentioned briefly were promoted for eliminating several complicated tasks at the workshop for business management.

11 a The politician that the doctor opposed adamantly was trusted after solving the health crisis himself at the conference for medical research.

11 b The politician that the organization opposed adamantly was trusted after solving the health crisis himself at the conference for medical research.

11 c The plan that the doctor opposed adamantly was trusted after solving the health crisis himself at the conference for medical research.

11 d The plan that the organization opposed adamantly was trusted after solving the health crisis himself at the conference for medical research.

11 e The politician that the doctor opposed adamantly was trusted after solving the health crisis at the conference for medical research.

11 f The politician that the organizations opposed adamantly was trusted after solving the health crisis at the conference for medical research.

11 g The plan that the doctors opposed adamantly were trusted after solving the health crisis at the conference for medical research.

11 h The plan that the organization opposed adamantly were trusted after solving the health crisis at the conference for medical research.

12 a The housewife that the maid disregarded blatantly was disliked for causing several major problems herself without any regard for minority populations.

12 b The housewife that the statement disregarded blatantly was disliked for causing several major problems herself without any regard for minority populations.

12 c The law that the maid disregarded blatantly was disliked for causing several major problems herself without any regard for minority populations.

12 d The law that the statement disregarded blatantly was disliked for causing several major problems herself without any regard for minority populations.

12 e The housewife that the maid disregarded blatantly was disliked for causing several major problems without any regard for minority populations.

12 f The housewife that the statements disregarded blatantly was disliked for causing several major problems without any regard for minority populations.

12 g The law that the maids disregarded blatantly were disliked for causing several major problems without any regard for minority populations.

12 h The law that the statement disregarded blatantly were disliked for causing several major problems without any regard for minority populations.

13 a The director that the critic advertised widely was acclaimed for showcasing the new movie himself without any funding from the executives.

13 b The director that the theater advertised widely was acclaimed for showcasing the new movie himself without any funding from the executives.

13 c The movie that the critic advertised widely was acclaimed for showcasing the new movie himself without any funding from the executives.

13 d The movie that the theater advertised widely was acclaimed for showcasing the new movie himself without any funding from the executives.

13 e The director that the critic advertised widely was acclaimed for showcasing the new movie without any funding from the executives.

13 f The director that the theaters advertised widely was acclaimed for showcasing the new movie without any funding from the executives.

13 g The movie that the critics advertised widely were acclaimed for showcasing the new movie without any funding from the executives.

13 h The movie that the theater advertised widely were acclaimed for showcasing the new movie without any funding from the executives.

14 a The marine that the officer discussed briefly was approved after resolving several major problems himself over the span of several days.

14 b The marine that the document discussed briefly was approved after resolving several major problems himself over the span of several days.

14 c The plan that the officer discussed briefly was approved after resolving several major problems himself over the span of several days.

14 d The plan that the document discussed briefly was approved after resolving several major problems himself over the span of several days.

14 e The marine that the officer discussed briefly was approved after resolving several major problems over the span of several days.

14 f The marine that the documents discussed briefly was approved after resolving several major problems over the span of several days.

14 g The plan that the officers discussed briefly were approved after resolving several major problems over the span of several days.

14 h The plan that the document discussed briefly were approved after resolving several major problems over the span of several days.

15 a The soldier that the officer recommended highly was praised for revealing the hidden agenda himself in the most subtle of ways.

15 b The soldier that the letter recommended highly was praised for revealing the hidden agenda himself in the most subtle of ways.

15 c The article that the officer recommended highly was praised for revealing the hidden agenda himself in the most subtle of ways.

15 d The article that the letter recommended highly was praised for revealing the hidden agenda himself in the most subtle of ways.

15 e The soldier that the officer recommended highly was praised for revealing the hidden agenda in the most subtle of ways.

15 f The soldier that the letters recommended highly was praised for revealing the hidden agenda in the most subtle of ways.

15 g The article that the officers recommended highly were praised for revealing the hidden agenda in the most subtle of ways.

15 h The article that the letter recommended highly were praised for revealing the hidden agenda in the most subtle of ways.

16 a The congresswoman that the counselor assessed critically was commended for reducing the tax rates herself without any increase in federal spending.

16 b The congresswoman that the proposal assessed critically was commended for reducing the tax rates herself without any increase in federal spending.

16 c The budget that the counselor assessed critically was commended for reducing the tax rates herself without any increase in federal spending.

16 d The budget that the proposal assessed critically was commended for reducing the tax rates herself without any increase in federal spending.

16 e The congresswoman that the counselor assessed critically was commended for reducing the tax rates without any increase in federal spending.

16 f The congresswoman that the proposals assessed critically was commended for reducing the tax rates without any increase in federal spending.

16 g The budget that the counselors assessed critically were commended for reducing the tax rates without any increase in federal spending.

16 h The budget that the proposal assessed critically were commended for reducing the tax rates without any increase in federal spending.

17 a The secretary that the librarian reviewed carefully was acclaimed for translating the lengthy letter herself without the use of fancy technology.

17 b The secretary that the article reviewed carefully was acclaimed for translating the lengthy letter herself without the use of fancy technology.

17 c The software that the librarian reviewed carefully was acclaimed for translating the lengthy letter herself without the use of fancy technology.

17 d The software that the article reviewed carefully was acclaimed for translating the lengthy letter herself without the use of fancy technology.

17 e The secretary that the librarian reviewed carefully was acclaimed for translating the lengthy letter without the use of fancy technology.

17 f The secretary that the articles reviewed carefully was acclaimed for translating the lengthy letter without the use of fancy technology.

17 g The software that the librarians reviewed carefully were acclaimed for translating the lengthy letter without the use of fancy technology.

17 h The software that the article reviewed carefully were acclaimed for translating the lengthy letter without the use of fancy technology.

18 a The dancer that the choreographer promoted widely was admired for portraying several important roles herself in the highly anticipated opening show.

18 b The dancer that the preview promoted widely was admired for portraying several important roles herself in the highly anticipated opening show.

18 c The musical that the choreographer promoted widely was admired for portraying several important roles herself in the highly anticipated opening show.

18 d The musical that the preview promoted widely was admired for portraying several important roles herself in the highly anticipated opening show.

18 e The dancer that the choreographer promoted widely was admired for portraying several important roles in the highly anticipated opening show.

18 f The dancer that the previews promoted widely was admired for portraying several important roles in the highly anticipated opening show.

18 g The musical that the choreographers promoted widely were admired for portraying several important roles in the highly anticipated opening show.

18 h The musical that the preview promoted widely were admired for portraying several important roles in the highly anticipated opening show.

19 a The carpenter that the executive authorized professionally was evaluated after establishing the first labor union himself without any funding from the board.

19 b The carpenter that the organization authorized professionally was evaluated after establishing the first labor union himself without any funding from the board.

19 c The program that the executive authorized professionally was evaluated after establishing the first labor union himself without any funding from the board.

19 d The program that the organization authorized professionally was evaluated after establishing the first labor union himself without any funding from the board.

19 e The carpenter that the executive authorized professionally was evaluated after establishing the first labor union without any funding from the board.

19 f The carpenter that the organizations authorized professionally was evaluated after establishing the first labor union without any funding from the board.

19 g The program that the executives authorized professionally were evaluated after establishing the first labor union without any funding from the board.

19 h The program that the organization authorized professionally were evaluated after establishing the first labor union without any funding from the board.

20 a The newsman that the editor disregarded blatantly was ignored after dispelling the horrible rumor himself without any confirmation from the staff.

20 b The newsman that the tabloid disregarded blatantly was ignored after dispelling the horrible rumor himself without any confirmation from the staff.

20 c The statement that the editor disregarded blatantly was ignored after dispelling the horrible rumor himself without any confirmation from the staff.

20 d The statement that the tabloid disregarded blatantly was ignored after dispelling the horrible rumor himself without any confirmation from the staff.

20 e The newsman that the editor disregarded blatantly was ignored after dispelling the horrible rumor without any confirmation from the staff.

20 f The newsman that the tabloids disregarded blatantly was ignored after dispelling the horrible rumor without any confirmation from the staff.

20 g The statement that the editors disregarded blatantly were ignored after dispelling the horrible rumor without any confirmation from the staff.

20 h The statement that the tabloid disregarded blatantly were ignored after dispelling the horrible rumor without any confirmation from the staff.

21 a The supermodel that the stylist praised highly was applauded for showcasing the fabulous wardrobe herself at the theater in the city.

21 b The supermodel that the commercial praised highly was applauded for showcasing the fabulous wardrobe herself at the theater in the city.

21 c The musical that the stylist praised highly was applauded for showcasing the fabulous wardrobe herself at the theater in the city.

21 d The musical that the commercial praised highly was applauded for showcasing the fabulous wardrobe herself at the theater in the city.

21 e The supermodel that the stylist praised highly was applauded for showcasing the fabulous wardrobe at the theater in the city.

21 f The supermodel that the commercials praised highly was applauded for showcasing the fabulous wardrobe at the theater in the city.

21 g The musical that the stylists praised highly were applauded for showcasing the fabulous wardrobe at the theater in the city.

21 h The musical that the commercial praised highly were applauded for showcasing the fabulous wardrobe at the theater in the city.

22 a The countess that the secretary supported dutifully was praised for financing the entire event herself without any donations from the public.

22 b The countess that the fund supported dutifully was praised for financing the entire event herself without any donations from the public.

22 c The organization that the secretary supported dutifully was praised for financing the entire event herself without any donations from the public.

22 d The organization that the fund supported dutifully was praised for financing the entire event herself without any donations from the public.

22 e The countess that the secretary supported dutifully was praised for financing the entire event without any donations from the public.

22 f The countess that the funds supported dutifully was praised for financing the entire event without any donations from the public.

22 g The organization that the secretaries supported dutifully were praised for financing the entire event without any donations from the public.

22 h The organization that the fund supported dutifully were praised for financing the entire event without any donations from the public.

23 a The statesman that the senator honored dutifully was appreciated for resolving the financial problem himself without any burden on the taxpayers.

23 b The statesman that the contract honored dutifully was appreciated for resolving the financial problem himself without any burden on the taxpayers.

23 c The law that the senator honored dutifully was appreciated for resolving the financial problem himself without any burden on the taxpayers.

23 d The law that the contract honored dutifully was appreciated for resolving the financial problem himself without any burden on the taxpayers.

23 e The statesman that the senator honored dutifully was appreciated for resolving the financial problem without any burden on the taxpayers.

23 f The statesman that the contracts honored dutifully was appreciated for resolving the financial problem without any burden on the taxpayers.

23 g The law that the senators honored dutifully were appreciated for resolving the financial problem without any burden on the taxpayers.

23 h The law that the contract honored dutifully were appreciated for resolving the financial problem without any burden on the taxpayers.

24 a The doctor that the researcher evaluated extensively was commended after disproving the controversial theory himself at the research institute in Europe.

24 b The doctor that the report evaluated extensively was commended after disproving the controversial theory himself at the research institute in Europe.

24 c The experiment that the researcher evaluated extensively was commended after disproving the controversial theory himself at the research institute in Europe.

24 d The experiment that the report evaluated extensively was commended after disproving the controversial theory himself at the research institute in Europe.

24 e The doctor that the researcher evaluated extensively was commended after disproving the controversial theory at the research institute in Europe.

24 f The doctor that the reports evaluated extensively was commended after disproving the controversial theory at the research institute in Europe.

24 g The experiment that the researchers evaluated extensively were commended after disproving the controversial theory at the research institute in Europe.

24 h The experiment that the report evaluated extensively were commended after disproving the controversial theory at the research institute in Europe.

25 a The congressman that the legislator disregarded boldly was denounced after introducing several unnecessary restrictions himself at the expense of the taxpayers.

25 b The congressman that the ruling disregarded boldly was denounced after introducing several unnecessary restrictions himself at the expense of the taxpayers.

25 c The policy that the legislator disregarded boldly was denounced after introducing several unnecessary restrictions himself at the expense of the taxpayers.

25 d The policy that the ruling disregarded boldly was denounced after introducing several unnecessary restrictions himself at the expense of the taxpayers.

25 e The congressman that the legislator disregarded boldly was denounced after introducing several unnecessary restrictions at the expense of the taxpayers.

25 f The congressman that the rulings disregarded boldly was denounced after introducing several unnecessary restrictions at the expense of the taxpayers.

25 g The policy that the legislators disregarded boldly were denounced after introducing several unnecessary restrictions at the expense of the taxpayers.

25 h The policy that the ruling disregarded boldly were denounced after introducing several unnecessary restrictions at the expense of the taxpayers.

26 a The sniper that the lawyer mentioned briefly was verified before describing the horrible crime himself in the very controversial court case.

26 b The sniper that the document mentioned briefly was verified before describing the horrible crime himself in the very controversial court case.

26 c The testimony that the lawyer mentioned briefly was verified before describing the horrible crime himself in the very controversial court case.

26 d The testimony that the document mentioned briefly was verified before describing the horrible crime himself in the very controversial court case.

26 e The sniper that the lawyer mentioned briefly was verified before describing the horrible crime in the very controversial court case.

26 f The sniper that the documents mentioned briefly was verified before describing the horrible crime in the very controversial court case.

26 g The testimony that the lawyers mentioned briefly were verified before describing the horrible crime in the very controversial court case.

26 h The testimony that the document mentioned briefly were verified before describing the horrible crime in the very controversial court case.

27 a The doctor that the researcher described meticulously was certified after debunking the urban myth himself in the new scientific journal article.

27 b The doctor that the report described meticulously was certified after debunking the urban myth himself in the new scientific journal article.

27 c The discovery that the researcher described meticulously was certified after debunking the urban myth himself in the new scientific journal article.

27 d The discovery that the report described meticulously was certified after debunking the urban myth himself in the new scientific journal article.

27 e The doctor that the researcher described meticulously was certified after debunking the urban myth in the new scientific journal article.

27 f The doctor that the reports described meticulously was certified after debunking the urban myth in the new scientific journal article.

27 g The discovery that the researchers described meticulously were certified after debunking the urban myth in the new scientific journal article.

27 h The discovery that the report described meticulously were certified after debunking the urban myth in the new scientific journal article.

28 a The actor that the critic mentioned repeatedly was nominated for portraying several important roles himself without the use of special effects.

28 b The actor that the preview mentioned repeatedly was nominated for portraying several important roles himself without the use of special effects.

28 c The film that the critic mentioned repeatedly was nominated for portraying several important roles himself without the use of special effects.

28 d The film that the preview mentioned repeatedly was nominated for portraying several important roles himself without the use of special effects.

28 e The actor that the critic mentioned repeatedly was nominated for portraying several important roles without the use of special effects.

28 f The actor that the previews mentioned repeatedly was nominated for portraying several important roles without the use of special effects.

28 g The film that the critics mentioned repeatedly were nominated for portraying several important roles without the use of special effects.

28 h The film that the preview mentioned repeatedly were nominated for portraying several important roles without the use of special effects.

29 a The smuggler that the lawyer evaluated quickly was dismissed after discrediting the slanderous claims himself in the court case on television.

29 b The smuggler that the investigation evaluated quickly was dismissed after discrediting the slanderous claims himself in the court case on television.

29 c The evidence that the lawyer evaluated quickly was dismissed after discrediting the slanderous claims himself in the court case on television.

29 d The evidence that the investigation evaluated quickly was dismissed after discrediting the slanderous claims himself in the court case on television.

29 e The smuggler that the lawyer evaluated quickly was dismissed after discrediting the slanderous claims in the court case on television.

29 f The smuggler that the investigations evaluated quickly was dismissed after discrediting the slanderous claims in the court case on television.

29 g The evidence that the lawyers evaluated quickly were dismissed after discrediting the slanderous claims in the court case on television.

29 h The evidence that the investigation evaluated quickly were dismissed after discrediting the slanderous claims in the court case on television.

30 a The lawyer that the officer addressed formally was dismissed after verifying the disputed testimony himself in the very controversial murder trial.

30 b The lawyer that the letter addressed formally was dismissed after verifying the disputed testimony himself in the very controversial murder trial.

30 c The evidence that the officer addressed formally was dismissed after verifying the disputed testimony himself in the very controversial murder trial.

30 d The evidence that the letter addressed formally was dismissed after verifying the disputed testimony himself in the very controversial murder trial.

30 e The lawyer that the officer addressed formally was dismissed after verifying the disputed testimony in the very controversial murder trial.

30 f The lawyer that the letters addressed formally was dismissed after verifying the disputed testimony in the very controversial murder trial.

30 g The evidence that the officers addressed formally were dismissed after verifying the disputed testimony in the very controversial murder trial.

30 h The evidence that the letter addressed formally were dismissed after verifying the disputed testimony in the very controversial murder trial.

31 a The protestor that the cop provoked suddenly was blamed for starting the massive riot himself in the typically peaceful French province.

31 b The protestor that the election provoked suddenly was blamed for starting the massive riot himself in the typically peaceful French province.

31 c The protest that the cop provoked suddenly was blamed for starting the massive riot himself in the typically peaceful French province.

31 d The protest that the election provoked suddenly was blamed for starting the massive riot himself in the typically peaceful French province.

31 e The protestor that the cop provoked suddenly was blamed for starting the massive riot in the typically peaceful French province.

31 f The protestor that the elections provoked suddenly was blamed for starting the massive riot in the typically peaceful French province.

31 g The protest that the cops provoked suddenly were blamed for starting the massive riot in the typically peaceful French province.

31 h The protest that the election provoked suddenly were blamed for starting the massive riot in the typically peaceful French province.

32 a The congressman that the representative assessed critically was opposed for authorizing the judicial appeal himself without the proper mandate from senate.

32 b The congressman that the article assessed critically was opposed for authorizing the judicial appeal himself without the proper mandate from senate.

32 c The legislation that the representative assessed critically was opposed for authorizing the judicial appeal himself without the proper mandate from senate.

32 d The legislation that the article assessed critically was opposed for authorizing the judicial appeal himself without the proper mandate from senate.

32 e The congressman that the representative assessed critically was opposed for authorizing the judicial appeal without the proper mandate from senate.

32 f The congressman that the articles assessed critically was opposed for authorizing the judicial appeal without the proper mandate from senate.

32 g The legislation that the representatives assessed critically were opposed for authorizing the judicial appeal without the proper mandate from senate.

32 h The legislation that the article assessed critically were opposed for authorizing the judicial appeal without a proper mandate from senate.

33 a The representative that the senator supported fully was opposed after eliminating the afterschool program himself due to a lack of funding.

33 b The representative that the university supported fully was opposed after eliminating the afterschool program himself due to a lack of funding.

33 c The contract that the senator supported fully was opposed after eliminating the afterschool program himself due to a lack of funding.

33 d The contract that the university supported fully was opposed after eliminating the afterschool program himself due to a lack of funding.

33 e The representative that the senator supported fully was opposed after eliminating the afterschool program due to a lack of funding.

33 f The representative that the universities supported fully was opposed after eliminating the afterschool program due to a lack of funding.

33 g The contract that the senators supported fully were opposed after eliminating the afterschool program due to a lack of funding.

33 h The contract that the university supported fully were opposed after eliminating the afterschool program due to a lack of funding.

34 a The architect that the executive referenced explicitly was certified after improving the preliminary design himself in response to the harsh criticisms.

34 b The architect that the document referenced explicitly was certified after improving the preliminary design himself in response to the harsh criticisms.

34 c The blueprint that the executive referenced explicitly was certified after improving the preliminary design himself in response to the harsh criticisms.

34 d The blueprint that the document referenced explicitly was certified after improving the preliminary design himself in response to the harsh criticisms.

34 e The architect that the executive referenced explicitly was certified after improving the preliminary design in response to the harsh criticisms.

34 f The architect that the documents referenced explicitly was certified after improving the preliminary design in response to the harsh criticisms.

34 g The blueprint that the executives referenced explicitly were certified after improving the preliminary design in response to the harsh criticisms.

34 h The blueprint that the document referenced explicitly were certified after improving the preliminary design in response to the harsh criticisms.

35 a The politician that the journalist critiqued harshly was favored for preventing the tax increase himself without any impact on the budget.

35 b The politician that the editorial critiqued harshly was favored for preventing the tax increase himself without any impact on the budget.

35 c The bill that the journalist critiqued harshly was favored for preventing the tax increase himself without any impact on the budget.

35 d The bill that the editorial critiqued harshly was favored for preventing the tax increase himself without any impact on the budget.

35 e The politician that the journalist critiqued harshly was favored for preventing the tax increase without any impact on the budget.

35 f The politician that the editorials critiqued harshly was favored for preventing the tax increase without any impact on the budget.

35 g The bill that the journalists critiqued harshly were favored for preventing the tax increase without any impact on the budget.

35 h The bill that the editorial critiqued harshly were favored for preventing the tax increase without any impact on the budget.

36 a The mathematician that the professor referenced explicitly was noted for deriving the correct answer himself without the use of obscure notation.

36 b The mathematician that the book referenced explicitly was noted for deriving the correct answer himself without the use of obscure notation.

36 c The algorithm that the professor referenced explicitly was noted for deriving the correct answer himself without the use of obscure notation.

36 d The algorithm that the book referenced explicitly was noted for deriving the correct answer himself without the use of obscure notation.

36 e The mathematician that the professor referenced explicitly was noted for deriving the correct answer without the use of obscure notation.

36 f The mathematician that the books referenced explicitly was noted for deriving the correct answer without the use of obscure notation.

36 g The algorithm that the professors referenced explicitly were noted for deriving the correct answer without the use of obscure notation.

36 h The algorithm that the book referenced explicitly were noted for deriving the correct answer without the use of obscure notation.

37 a The protestor that the historian analyzed critically was commended for starting the political movement himself in reaction to the unfair law.

37 b The protestor that the book analyzed critically was commended for starting the political movement himself in reaction to the unfair law.

37 c The manifesto that the historian analyzed critically was commended for starting the political movement himself in reaction to the unfair law.

37 d The manifesto that the book analyzed critically was commended for starting the political movement himself in reaction to the unfair law.

37 e The protestor that the historian analyzed critically was commended for starting the political movement in reaction to the unfair law.

37 f The protestor that the books analyzed critically was commended for starting the political movement in reaction to the unfair law.

37 g The manifesto that the historians analyzed critically were commended for starting the political movement in reaction to the unfair law.

37 h The manifesto that the book analyzed critically were commended for starting the political movement in reaction to the unfair law.

38 a The congressman that the senator referenced explicitly was respected for confirming the election results himself in a timely and efficient manner.

38 b The congressman that the broadcast referenced explicitly was respected for confirming the election results himself in a timely and efficient manner.

38 c The newspaper that the senator referenced explicitly was respected for confirming the election results himself in a timely and efficient manner.

38 d The newspaper that the broadcast referenced explicitly was respected for confirming the election results himself in a timely and efficient manner.

38 e The congressman that the senator referenced explicitly was respected for confirming the election results in a timely and efficient manner.

38 f The congressman that the broadcasts referenced explicitly was respected for confirming the election results in a timely and efficient manner.

38 g The newspaper that the senators referenced explicitly were respected for confirming the election results in a timely and efficient manner.

38 h The newspaper that the broadcast referenced explicitly were respected for confirming the election results in a timely and efficient manner.

39 a The mathematician that the professor mentioned repeatedly was referenced for deriving the correct solution himself in a simple and elegant way.

39 b The mathematician that the textbook mentioned repeatedly was referenced for deriving the correct solution himself in a simple and elegant way.

39 c The equation that the professor mentioned repeatedly was referenced for deriving the correct solution himself in a simple and elegant way.

39 d The equation that the textbook mentioned repeatedly was referenced for deriving the correct solution himself in a simple and elegant way.

39 e The mathematician that the professor mentioned repeatedly was referenced for deriving the correct solution in a simple and elegant way.

39 f The mathematician that the textbooks mentioned repeatedly was referenced for deriving the correct solution in a simple and elegant way.

39 g The equation that the professors mentioned repeatedly were referenced for deriving the correct solution in a simple and elegant way.

39 h The equation that the textbook mentioned repeatedly were referenced for deriving the correct solution in a simple and elegant way.

40 a The schoolgirl that the nurse certified formally was appreciated for completing many important tasks herself in a short period of time.

40 b The schoolgirl that the workshop certified formally was appreciated for completing many important tasks herself in a short period of time.

40 c The tool that the nurse certified formally was appreciated for completing many important tasks herself in a short period of time.

40 d The tool that the workshop certified formally was appreciated for completing many important tasks herself in a short period of time.

40 e The schoolgirl that the nurse certified formally was appreciated for completing many important tasks in a short period of time.

40 f The schoolgirl that the workshops certified formally was appreciated for completing many important tasks in a short period of time.

40 g The tool that the nurses certified formally were appreciated for completing many important tasks in a short period of time.

40 h The tool that the workshop certified formally were appreciated for completing many important tasks in a short period of time.

41 a The officer that the lawyer described accurately was reviewed after revealing the crucial evidence himself in a report for the government.

41 b The officer that the letter described accurately was reviewed after revealing the crucial evidence himself in a report for the government.

41 c The investigation that the lawyer described accurately was reviewed after revealing the crucial evidence himself in a report for the government.

41 d The investigation that the letter described accurately was reviewed after revealing the crucial evidence himself in a report for the government.

41 e The officer that the lawyer described accurately was reviewed after revealing the crucial evidence in a report for the government.

41 f The officer that the letters described accurately was reviewed after revealing the crucial evidence in a report for the government.

41 g The investigation that the lawyers described accurately were reviewed after revealing the crucial evidence in a report for the government.

41 h The investigation that the letter described accurately were reviewed after revealing the crucial evidence in a report for the government.

42 a The landlord that the janitor recommended highly was certified for eradicating the filthy rats himself at the very large apartment complex.

42 b The landlord that the service recommended highly was certified for eradicating the filthy rats himself at the very large apartment complex.

42 c The chemical that the janitor recommended highly was certified for eradicating the filthy rats himself at the very large apartment complex.

42 d The chemical that the service recommended highly was certified for eradicating the filthy rats himself at the very large apartment complex.

42 e The landlord that the janitor recommended highly was certified for eradicating the filthy rats at the very large apartment complex.

42 f The landlord that the services recommended highly was certified for eradicating the filthy rats at the very large apartment complex.

42 g The chemical that the janitors recommended highly were certified for eradicating the filthy rats at the very large apartment complex.

42 h The chemical that the service recommended highly were certified for eradicating the filthy rats at the very large apartment complex.

43 a The housewife that the maid described carefully was deplored for causing the unnecessary drama herself in the quite community across town.

43 b The housewife that the file described carefully was deplored for causing the unnecessary drama herself in the quite community across town.

43 c The crime that the maid described carefully was deplored for causing the unnecessary drama herself in the quite community across town.

43 d The crime that the file described carefully was deplored for causing the unnecessary drama herself in the quite community across town.

43 e The housewife that the maid described carefully was deplored for causing the unnecessary drama in the quite community across town.

43 f The housewife that the files described carefully was deplored for causing the unnecessary drama in the quite community across town.

43 g The crime that the maids described carefully were deplored for causing the unnecessary drama in the quite community across town.

43 h The crime that the file described carefully were deplored for causing the unnecessary drama in the neighborhood outside the city.

44 a The newswoman that the secretary interrupted briefly was applauded for setting the new fashion trend herself without the use of expensive makeup.

44 b The newswoman that the commercial interrupted briefly was applauded for setting the new fashion trend herself without the use of expensive makeup.

44 c The show that the secretary interrupted briefly was applauded for setting the new fashion trend herself without the use of expensive makeup.

44 d The show that the commercial interrupted briefly was applauded for setting the new fashion trend herself without the use of expensive makeup.

44 e The newswoman that the secretary interrupted briefly was applauded for setting the new fashion trend without the use of expensive makeup.

44 f The newswoman that the commercials interrupted briefly was applauded for setting the new fashion trend without the use of expensive makeup.

44 g The show that the secretaries interrupted briefly were applauded for setting the new fashion trend without the use of expensive makeup.

44 h The show that the commercial interrupted briefly were applauded for setting the new fashion trend without the use of expensive makeup.

45 a The receptionist that the stylist contradicted blatantly was trusted after verifying the harsh accusations herself in the very controversial court case.

45 b The receptionist that the statement contradicted blatantly was trusted after verifying the harsh accusations herself in the very controversial court case.

45 c The testimony that the stylist contradicted blatantly was trusted after verifying the harsh accusations herself in the very controversial court case.

45 d The testimony that the statement contradicted blatantly was trusted after verifying the harsh accusations herself in the very controversial court case.

45 e The receptionist that the stylist contradicted blatantly was trusted after verifying the harsh accusations in the very controversial court case.

45 f The receptionist that the statements contradicted blatantly was trusted after verifying the harsh accusations in the very controversial court case.

45 g The testimony that the stylists contradicted blatantly were trusted after verifying the harsh accusations in the very controversial court case.

45 h The testimony that the statement contradicted blatantly were trusted after verifying the harsh accusations in the very controversial court case.

46 a The engineer that the executive noted officially was approved for authorizing the large purchase himself at the warehouse by the river.

46 b The engineer that the certificate noted officially was approved for authorizing the large purchase himself at the warehouse by the river.

46 c The document that the executive noted officially was approved for authorizing the large purchase himself at the warehouse by the river.

46 d The document that the certificate noted officially was approved for authorizing the large purchase himself at the warehouse by the river.

46 e The engineer that the executive noted officially was approved for authorizing the large purchase at the warehouse by the river.

46 f The engineer that the certificates noted officially was approved for authorizing the large purchase at the warehouse by the river.

46 g The document that the executives noted officially were approved for authorizing the large purchase at the warehouse by the river.

46 h The document that the certificate noted officially were approved for authorizing the large purchase at the warehouse by the river.

47 a The girlscout that the counselor praised highly was noted for developing the new procedure herself at the school for gifted students.

47 b The girlscout that the newspaper praised highly was noted for developing the new procedure herself at the school for gifted students.

47 c The project that the counselor praised highly was noted for developing the new procedure herself at the school for gifted students.

47 d The project that the newspaper praised highly was noted for developing the new procedure herself at the school for gifted students.

47 e The girl scout that the counselor praised highly was noted for developing the new procedure at the school for gifted students.

47 f The girl scout that the newspapers praised highly was noted for developing the new procedure at the school for gifted students.

47 g The project that the counselors praised highly were noted for developing the new procedure at the school for gifted students.

47 h The project that the newspaper praised highly were noted for developing the new procedure at the school for gifted students.

48 a The builder that the administrator approved quickly was confirmed before detecting several major problems himself in the design for the structure.

48 b The builder that the corporation approved quickly was confirmed before detecting several major problems himself in the design for the structure.

48 c The inspection that the administrator approved quickly was confirmed before detecting several major problems himself in the design for the structure.

48 d The inspection that the corporation approved quickly was confirmed before detecting several major problems himself in the design for the structure.

48 e The builder that the administrator approved quickly was confirmed before detecting several major problems in the design for the structure.

48 f The builder that the corporations approved quickly was confirmed before detecting several major problems in the design for the structure.

48 g The inspection that the administrators approved quickly were confirmed before detecting several major problems in the design for the structure.

48 h The inspection that the corporation approved quickly were confirmed before detecting several major problems in the design for the structure.

Items from Experiment 3

a Adjunct control: Grammatical, distractor

b Adjunct control: Grammatical, no distractor

c Adjunct control: Ungrammatical, distractor

d Adjunct control: Ungrammatical, no distractor

e Subject-verb agreement: Grammatical, distractor

f Subject-verb agreement: Grammatical, no distractor

g Subject-verb agreement: Ungrammatical, distractor

h Subject-verb agreement: Ungrammatical, no distractor

1 a The spokeswoman that the secretary greeted at the front_desk was praised after fixing the broken scale herself without any help from the technician.

1 b The spokeswoman that the doctor greeted at the front_desk was praised after fixing the broken scale herself without any help from the technician.

1 c The surgeon that the nurse greeted at the front_desk was praised after fixing the broken scale herself without any help from the technician.

1 d The surgeon that the doctor greeted at the front_desk was praised after fixing the broken scale herself without any help from the technician.

1 e The spokeswoman that the nurse greeted at the front_desk was praised after fixing the broken scale without any help from the technician.

1 f The spokeswoman that the doctors greeted at the front_desk was praised after fixing the broken scale without any help from the technician.

1 g The surgeon that the nurses greeted at the front_desk were praised after fixing the broken scale without any help from the technician.

1 h The surgeon that the doctor greeted at the front_desk were praised after fixing the broken scale without any help from the technician.

2 a The harpist that the diva liked very much was congratulated after playing the beautiful song herself at the brand new recording studio.

2 b The harpist that the guitarist liked very much was congratulated after playing the beautiful song herself at the brand new recording studio.

2 c The drummer that the diva liked very much was congratulated after playing the beautiful song herself at the brand new recording studio.

2 d The drummer that the guitarist liked very much was congratulated after playing the beautiful song herself at the brand new recording studio.

2 e The harpist that the diva liked very much was congratulated after playing the beautiful song at the brand new recording studio.

2 f The harpist that the guitarists liked very much was congratulated after playing the beautiful song at the brand new recording studio.

2 g The drummer that the divas liked very much were congratulated after playing the beautiful song at the brand new recording studio.

2 h The drummer that the guitarist liked very much were congratulated after playing the beautiful song at the brand new recording studio.

3 a The cheerleader that the sorority_girl brought to the party was interrupted before revealing the dark secret herself about the affair with the professor.

3 b The cheerleader that the frat_boy brought to the party was interrupted before revealing the dark secret herself about the affair with the professor.

3 c The football_player that the sorority_girl brought to the party was interrupted before revealing the dark secret herself about the affair with the professor.

3 d The football_player that the frat_boy brought to the party was interrupted before revealing the dark secret herself about the affair with the professor.

3 e The cheerleader that the sorority_girl brought to the party was interrupted before revealing the dark secret about the affair with the professor.

3 f The cheerleader that the frat_boys brought to the party was interrupted before revealing the dark secret about the affair with the professor.

3 g The football player that the sorority_girls brought to the party were interrupted before revealing the dark secret about the affair with the professor.

3 h The football player that the frat_boy brought to the party were interrupted before revealing the dark secret about the affair with the professor.

4 a The hairdresser that the manicurist recommended very enthusiastically was questioned before accepting all the blame herself for the mistake with the bill.

4 b The hairdresser that the manager recommended very enthusiastically was questioned before accepting all the blame herself for the mistake with the bill.

4 c The barber that the manicurist recommended very enthusiastically was questioned before accepting all the blame herself for the mistake with the bill.

4 d The barber that the manager recommended very enthusiastically was questioned before accepting all the blame herself for the mistake with the bill.

4 e The hairdresser that the manicurist recommended very enthusiastically was questioned before accepting all the blame for the mistake with the bill.

4 f The hairdresser that the managers recommended very enthusiastically was questioned before accepting all the blame for the mistake with the bill.

4 g The barber that the manicurists recommended very enthusiastically were questioned before accepting all the blame for the mistake with the bill.

4 h The barber that the manager recommended very enthusiastically were questioned before accepting all the blame for the mistake with the bill.

5 a The florist that the bridesmaid hired for the wedding was greeted before setting up some decorations herself for the large and expensive ceremony.

5 b The florist that the groomsman hired for the wedding was greeted before setting up some decorations herself for the large and expensive ceremony.

5 c The bartender that the bridesmaid hired for the wedding was greeted before setting up some decorations herself for the large and expensive ceremony.

5 d The bartender that the groomsman hired for the wedding was greeted before setting up some decorations herself for the large and expensive ceremony.

5 e The florist that the bridesmaid hired for the wedding was greeted before setting up some decorations herself for the large and expensive ceremony.

5 f The florist that the groomsmen hired for the wedding was greeted before setting up some decorations herself for the large and expensive ceremony.

5 g The bartender that the bridesmaids hired for the wedding were greeted before setting up some decorations herself for the large and expensive ceremony.

5 h The bartender that the groomsman hired for the wedding were greeted before setting up some decorations herself for the large and expensive ceremony.

6 a The congresswoman that the secretary disliked very much was rejected before contacting the local news herself about the false accusations on TV.

6 b The congresswoman that the activist disliked very much was rejected before contacting the local news herself about the false accusations on TV.

6 c The lobbyist that the secretary disliked very much was rejected before contacting the local news herself about the false accusations on TV.

6 d The lobbyist that the activist disliked very much was rejected before contacting the local news herself about the false accusations on TV.

6 e The congresswoman that the secretary disliked very much was rejected before contacting the local news about the false accusations on TV.

6 f The congresswoman that the activists disliked very much was rejected before contacting the local news about the false accusations on TV.

6 g The lobbyist that the secretaries disliked very much were rejected before contacting the local news about the false accusations on TV.

6 h The lobbyist that the activist disliked very much were rejected before contacting the local news about the false accusations on TV.

7 a The cleaning_lady that the nanny knew from high_school was reprimanded before cleaning the large mess herself without a mop from the closet.

7 b The cleaning_lady that the manager knew from high_school was reprimanded before cleaning the large mess herself without a mop from the closet.

7 c The chef that the nanny knew from high_school was reprimanded before cleaning the large mess herself without a mop from the closet.

7 d The chef that the manager knew from high_school was reprimanded before cleaning the large mess herself without a mop from the closet.

7 e The cleaning_lady that the nanny knew from high_school was reprimanded before cleaning the large mess without a mop from the closet.

7 f The cleaning_lady that the managers knew from high_school was reprimanded before cleaning the large mess without a mop from the closet.

7 g The chef that the nannies knew from high_school were reprimanded before cleaning the large mess without a mop from the closet.

7 h The chef that the manager knew from high_school were reprimanded before cleaning the large mess without a mop from the closet.

8 a The cleaning_lady that the landlady hired over the phone was interrupted before explaining all the charges herself on the bill for the job.

8 b The cleaning_lady that the landlord hired over the phone was interrupted before explaining all the charges herself on the bill for the job.

8 c The plumber that the landlady hired over the phone was interrupted before explaining all the charges herself on the bill for the job.

8 d The plumber that the landlord hired over the phone was interrupted before explaining all the charges herself on the bill for the job.

8 e The cleaning_lady that the landlady hired over the phone was interrupted before explaining all the charges on the bill for the job.

8 f The cleaning_lady that the landlords hired over the phone was interrupted before explaining all the charges on the bill for the job.

8 g The plumber that the landladies hired over the phone were interrupted before explaining all the charges on the bill for the job.

8 h The plumber that the landlord hired over the phone were interrupted before explaining all the charges on the bill for the job.

9 a The secretary that the saleswoman contacted through email was confronted before informing the sales department herself about the incident in the lobby.

9 b The secretary that the salesman contacted through email was confronted before informing the sales department herself about the incident in the lobby.

9 c The representative that the saleswoman contacted through email was confronted before informing the sales department herself about the incident in the lobby.

9 d The representative that the salesman contacted through email was confronted before informing the sales department herself about the incident in the lobby.

9 e The secretary that the saleswoman contacted through email was confronted before informing the sales department about the incident in the lobby.

9 f The secretary that the salesmen contacted through email was confronted before informing the sales department about the incident in the lobby.

9 g The representative that the saleswomen contacted through email were confronted before informing the sales department about the incident in the lobby.

9 h The representative that the salesman contacted through email were confronted before informing the sales department about the incident in the lobby.

10 a The librarian that the schoolgirl saw in the hall was questioned before reviewing the correct answers herself with the brand new computer program.

10 b The librarian that the schoolboy saw in the hall was questioned before reviewing the correct answers herself with the brand new computer program.

10 c The principal that the schoolgirl saw in the hall was questioned before reviewing the correct answers herself with the brand new computer program.

10 d The principal that the schoolboy saw in the hall was questioned before reviewing the correct answers herself with the brand new computer program.

10 e The librarian that the schoolgirl saw in the hall was questioned before reviewing the correct answers with the brand new computer program.

10 f The librarian that the schoolboys saw in the hall was questioned before reviewing the correct answers with the brand new computer program.

10 g The principal that the schoolgirls saw in the hall were questioned before reviewing the correct answers with the brand new computer program.

10 h The principal that the schoolboy saw in the hall were questioned before reviewing the correct answers with the brand new computer program.

11 a The religious_woman that the nun visited once a week was notified before checking the email message herself in the office for the church.

11 b The religious_woman that the monk visited once a week was notified before checking the email message herself in the office for the church.

11 c The priest that the nun visited once a week was notified before checking the email message herself in the office for the church.

11 d The priest that the monk visited once a week was notified before checking the email message herself in the office for the church.

11 e The religious_woman that the nun visited once a week was notified before checking the email message in the office for the church.

11 f The religious_woman that the monks visited once a week was notified before checking the email message in the office for the church.

11 g The priest that the nuns visited once a week were notified before checking the email message in the office for the church.

11 h The priest that the monk visited once a week were notified before checking the email message in the office for the church.

12 a The hostess that the waitress assisted at the restaurant was reprimanded before reporting the health violation herself to the editor of the newspaper.

12 b The hostess that the chef assisted at the restaurant was reprimanded before reporting the health violation herself to the editor of the newspaper.

12 c The busboy that the waitress assisted at the restaurant was reprimanded before reporting the health violation herself to the editor of the newspaper.

12 d The busboy that the chef assisted at the restaurant was reprimanded before reporting the health violation herself to the editor of the newspaper.

12 e The hostess that the waitress assisted at the restaurant was reprimanded before reporting the health violation to the editor of the newspaper.

12 f The hostess that the chefs assisted at the restaurant was reprimanded before reporting the health violation to the editor of the newspaper.

12 g The busboy that the waitresses assisted at the restaurant were reprimanded before reporting the health violation to the editor of the newspaper.

12 h The busboy that the chef assisted at the restaurant were reprimanded before reporting the health violation to the editor of the newspaper.

13 a The actress that the makeup_artist greeted at the studio was fired before calling the tabloid magazine herself about the horrible and malicious rumor.

13 b The actress that the director greeted at the studio was fired before calling the tabloid magazine herself about the horrible and malicious rumor.

13 c The actor that the makeup_artist greeted at the studio was fired before calling the tabloid magazine herself about the horrible and malicious rumor.

13 d The actor that the director greeted at the studio was fired before calling the tabloid magazine herself about the horrible and malicious rumor.

13 e The actress that the makeup_artist greeted at the studio was fired before calling the tabloid magazine about the horrible and malicious rumor.

13 f The actress that the directors greeted at the studio was fired before calling the tabloid magazine about the horrible and malicious rumor.

13 g The actor that the makeup_artists greeted at the studio were fired before calling the tabloid magazine about the horrible and malicious rumor.

13 h The actor that the director greeted at the studio were fired before calling the tabloid magazine about the horrible and malicious rumor.

14 a The gymnast that the ballerina mocked in the locker_room was criticized before leaking the important news herself to the journalist for the newspaper.

14 b The gymnast that the baseball player mocked in the locker_room was criticized before leaking the important news herself to the journalist for the newspaper.

14 c The coach that the ballerina mocked in the locker_room was criticized before leaking the important news herself to the journalist for the newspaper.

14 d The coach that the baseball player mocked in the locker_room was criticized before leaking the important news herself to the journalist for the newspaper.

14 e The gymnast that the ballerina mocked in the locker_room was criticized before leaking the important news to the journalist for the newspaper.

14 f The gymnast that the baseball players mocked in the locker_room was criticized before leaking the important news to the journalist for the newspaper.

14 g The coach that the ballerinas mocked in the locker_room were criticized before leaking the important news to the journalist for the newspaper.

14 h The coach that the baseball player mocked in the locker_room were criticized before leaking the important news to the journalist for the newspaper.

15 a The secretary that the businesswoman saw every morning was criticized before starting the malicious rumor herself during lunch in the corporate office.

15 b The secretary that the businessman saw every morning was criticized before starting the malicious rumor herself during lunch in the corporate office.

15 c The administrator that the businesswoman saw every morning was criticized before starting the malicious rumor herself during lunch in the corporate office.

15 d The administrator that the businessman saw every morning was criticized before starting the malicious rumor herself during lunch in the corporate office.

15 e The secretary that the businesswoman saw every morning was criticized before starting the malicious rumor during lunch in the corporate office.

15 f The secretary that the businessmen saw every morning was criticized before starting the malicious rumor during lunch in the corporate office.

15 g The administrator that the businesswomen saw every morning were criticized before starting the malicious rumor during lunch in the corporate office.

15 h The administrator that the businessman saw every morning were criticized before starting the malicious rumor during lunch in the corporate office.

16 a The cleaning_lady that the maid welcomed at the door was praised after removing the large stain herself in just a matter of minutes

16 b The cleaning_lady that the homeowner welcomed at the door was praised after removing the large stain herself in just a matter of minutes

16 c The plumber that the maid welcomed at the door was praised after removing the large stain herself in just a matter of minutes

16 d The plumber that the homeowner welcomed at the door was praised after removing the large stain herself in just a matter of minutes

16 e The cleaning_lady that the maid welcomed at the door was praised after removing the large stain in just a matter of minutes

16 f The cleaning_lady that the homeowners welcomed at the door was praised after removing the large stain in just a matter of minutes

16 g The plumber that the maids welcomed at the door were praised after removing the large stain in just a matter of minutes

16 h The plumber that the homeowners welcomed at the door were praised after removing the large stain in just a matter of minutes

17 a The single_mother that the stewardess offended at the airport was compensated after filing the complaint form herself at the headquarters of the airline.

17 b The single_mother that the businessman offended at the airport was compensated after filing the complaint form herself at the headquarters of the airline.

17 c The pilot that the stewardess offended at the airport was compensated after filing the complaint form herself at the headquarters of the airline.

17 d The pilot that the businessman offended at the airport was compensated after filing the complaint form herself at the headquarters of the airline.

17 e The single_mother that the stewardess offended at the airport was compensated after filing the complaint form at the headquarters of the airline.

17 f The single_mother that the businessmen offended at the airport was compensated after filing the complaint form at the headquarters of the airline.

17 g The pilot that the stewardesses offended at the airport were compensated after filing the complaint form at the headquarters of the airline.

17 h The pilot that the businessman offended at the airport were compensated after filing the complaint form at the headquarters of the airline.

18 a The diva that the fashion_model met at the concert was interviewed after calling the state police herself about the paparazzi in the building

18 b The diva that the talent_agent met at the concert was interviewed after calling the state police herself about the paparazzi in the building

18 c The rock_star that the fashion_model met at the concert was interviewed after calling the state police herself about the paparazzi in the building

18 d The rock_star that the talent_agent met at the concert was interviewed after calling the state police herself about the paparazzi in the building

18 e The diva that the fashion_model met at the concert was interviewed after calling the state police about the paparazzi in the building

18 f The diva that the talent_agents met at the concert was interviewed after calling the state police about the paparazzi in the building

18 g The rock star that the fashion_models met at the concert were interviewed after calling the state police about the paparazzi in the building

18 h The rock star that the talent_agent met at the concert were interviewed after calling the state police about the paparazzi in the building

19 a The actress that the hairdresser complimented very kindly was commended after paying for the event herself without any donations from the public.

19 b The actress that the cameraman complimented very kindly was commended after paying for the event herself without any donations from the public.

19 c The actor that the hairdresser complimented very kindly was commended after paying for the event herself without any donations from the public.

19 d The actor that the cameraman complimented very kindly was commended after paying for the event herself without any donations from the public.

19 e The actress that the hairdresser complimented very kindly was commended after paying for the event without any donations from the public.

19 f The actress that the cameramen complimented very kindly was commended after paying for the event without any donations from the public.

19 g The actor that the hairdressers complimented very kindly were commended after paying the entire event without any donations from the public.

19 h The actor that the cameraman complimented very kindly were commended after paying the entire event without any donations from the public.

20 a The nurse that the secretary helped during the procedure was commended after warning the hospital staff herself about the wheelchair that was broken.

20 b The nurse that the surgeon helped during the procedure was commended after warning the hospital staff herself about the wheelchair that was broken.

20 c The doctor that the secretary helped during the procedure was commended after warning the hospital staff herself about the wheelchair that was broken.

20 d The doctor that the surgeon helped during the procedure was commended after warning the hospital staff herself about the wheelchair that was broken.

20 e The nurse that the secretary helped during the procedure was commended after warning the hospital staff about the wheelchair that was broken.

20 f The nurse that the surgeons helped during the procedure was commended after warning the hospital staff about the wheelchair that was broken.

20 g The doctor that the secretaries helped during the procedure were commended after warning the hospital staff about the wheelchair that was broken.

20 h The doctor that the surgeon helped during the procedure were commended after warning the hospital staff about the wheelchair that was broken.

21 a The shopper that the saleswoman assisted at the store was impressed after testing the new product herself even though it was very expensive.

21 b The shopper that the salesman assisted at the store was impressed after testing the new product herself even though it was very expensive.

21 c The manager that the saleswoman assisted at the store was impressed after testing the new product herself even though it was very expensive.

21 d The manager that the salesman assisted at the store was impressed after testing the new product herself even though it was very expensive.

21 e The shopper that the saleswoman assisted at the store was impressed after testing the new product even though it was very expensive.

21 f The shopper that the salesmen assisted at the store was impressed after testing the new product even though it was very expensive.

21 g The manager that the saleswomen assisted at the store were impressed after testing the new product even though it was very expensive.

21 h The manager that the salesman assisted at the store were impressed after testing the new product even though it was very expensive.

22 a The file_clerk that the social_worker hired at the office was promoted after proofreading the entire report herself several days before the final deadline.

22 b The file_clerk that the chairman hired at the office was promoted after proofreading the entire report herself several days before the final deadline.

22 c The administrator that the social_worker hired at the office was promoted after proofreading the entire report herself several days before the final deadline.

22 d The administrator that the chairman hired at the office was promoted after proofreading the entire report herself several days before the final deadline.

22 e The file_clerk that the social_worker hired at the office was promoted after proofreading the entire report several days before the final deadline.

22 f The file_clerk that the chairmen hired at the office was promoted after proofreading the entire report several days before the final deadline.

22 g The administrator that the social_workers hired at the office were promoted after proofreading the entire report several days before the final deadline.

22 h The administrator that the chairman hired at the office were promoted after proofreading the entire report several days before the final deadline.

23 a The clerical_assistant that the caregiver advised over the phone was paged after picking up the prescription herself from the pharmacy at the hospital

23 b The clerical_assistant that the physician advised over the phone was paged after picking up the prescription herself from the pharmacy at the hospital

23 c The medic that the caregiver advised over the phone was paged after picking up the prescription herself from the pharmacy at the hospital

23 d The medic that the physician advised over the phone was paged after picking up the prescription herself from the pharmacy at the hospital

23 e The clerical_assistant that the caregiver advised over the phone was paged after picking up the prescription from the pharmacy at the hospital

23 f The clerical_assistant that the physicians advised over the phone was paged after picking up the prescription from the pharmacy at the hospital

23 g The medic that the caregivers advised over the phone was paged after picking up the prescription from the pharmacy at the hospital

23 h The medic that the physician advised over the phone was paged after picking up the prescription from the pharmacy at the hospital

24 a The file_clerk that the congresswoman praised very kindly was promoted after translating the long documents herself without the use of a dictionary.

24 b The file_clerk that the congressman praised very kindly was promoted after translating the long documents herself without the use of a dictionary.

24 c The errand_boy that the congresswoman praised very kindly was promoted after translating the long documents herself without the use of a dictionary.

24 d The errand_boy that the congressman praised very kindly was promoted after translating the long documents herself without the use of a dictionary.

24 e The file_clerk that the congresswoman praised very kindly was promoted after translating the long documents without the use of a dictionary.

24 f The file_clerk that the congressmen praised very kindly was promoted after translating the long documents without the use of a dictionary.

24 g The errand_boy that the congresswomen praised very kindly were promoted after translating the long documents without the use of a dictionary.

24 h The errand_boy that the congressman praised very kindly were promoted after translating the long documents without the use of a dictionary.

25 a The salesman that the executive despised at work was reprimanded before answering all the questions himself about the money that was missing.

25 b The salesman that the receptionist despised at work was reprimanded before answering all the questions himself about the money that was missing.

25 c The saleswoman that the executive despised at work was reprimanded before answering all the questions himself about the money that was missing.

25 d The saleswoman that the receptionist despised at work was reprimanded before answering all the questions himself about the money that was missing.

25 e The salesman that the executive despised at work was reprimanded before answering all the questions about the money that was missing.

25 f The salesman that the receptionists despised at work was reprimanded before answering all the questions about the money that was missing.

25 g The saleswoman that the executives despised at work were reprimanded before answering all the questions about the money that was missing.

25 h The saleswoman that the receptionist despised at work were reprimanded before answering all the questions about the money that was missing.

26 a The chef that the butcher welcomed at the front_door was complimented after preparing the extravagant meal himself in the kitchen at the restaurant.

26 b The chef that the hostess welcomed at the front_door was complimented after preparing the extravagant meal himself in the kitchen at the restaurant.

26 c The waitress that the butcher welcomed at the front_door was complimented after preparing the extravagant meal himself in the kitchen at the restaurant.

26 d The waitress that the hostess welcomed at the front_door was complimented after preparing the extravagant meal himself in the kitchen at the restaurant.

26 e The chef that the butcher welcomed at the front_door was complimented after preparing the extravagant meal in the kitchen at the restaurant.

26 f The chef that the hostesses welcomed at the front_door was complimented after preparing the extravagant meal in the kitchen at the restaurant.

26 g The waitress that the butchers welcomed at the front_door were complimented after preparing the extravagant meal in the kitchen at the restaurant.

26 h The waitress that the hostess welcomed at the front_door were complimented after preparing the extravagant meal in the kitchen at the restaurant.

27 a The lobbyist that the newsman questioned about the election was criticized before setting the record straight himself during the press televised political debate.

27 b The lobbyist that the newswoman questioned about the election was criticized before setting the record straight himself during the press televised political debate.

27 c The congresswoman that the newsman questioned about the election was criticized before setting the record straight himself during the press televised political debate.

27 d The congresswoman that the newswoman questioned about the election was criticized before setting the record straight himself during the press televised political debate.

27 e The lobbyist that the newsman questioned about the election was criticized before setting the record straight during the press televised political debate.

27 f The lobbyist that the newswomen questioned about the election was criticized before setting the record straight during the press televised political debate.

27 g The congresswoman that the newsmen questioned about the election were criticized before setting the record straight during the press televised political debate.

27 h The congresswoman that the newswoman questioned about the election were criticized before setting the record straight during the press televised political debate.

28 a The hiker that the woodsman rescued in the forest was interrupted before telling the heroic story himself around the campfire by the cabin.

28 b The hiker that the girlscout rescued in the forest was interrupted before telling the heroic story himself around the campfire by the cabin.

28 c The elderly_woman that the woodsman rescued in the forest was interrupted before telling the heroic story himself around the campfire by the cabin.

28 d The elderly_woman that the girlscout rescued in the forest was interrupted before telling the heroic story himself around the campfire by the cabin.

28 e The hiker that the woodsman rescued in the forest was interrupted before telling the heroic story around the campfire by the cabin.

28 f The hiker that the girlscouts rescued in the forest was interrupted before telling the heroic story around the campfire by the cabin.

28 g The elderly_woman that the woodsmen rescued in the forest were interrupted before telling the heroic story around the campfire by the cabin.

28 h The elderly_woman that the girlscout rescued in the forest were interrupted before telling the heroic story around the campfire by the cabin.

29 a The quarterback that the coach saw in the hall was criticized before explaining the awkward situation himself to the principal of the school.

29 b The quarterback that the cheerleader saw in the hall was criticized before explaining the awkward situation himself to the principal of the school.

29 c The gymnast that the coach saw in the hall was criticized before explaining the awkward situation himself to the principal of the school.

29 d The gymnast that the cheerleader saw in the hall was criticized before explaining the awkward situation himself to the principal of the school.

29 e The quarterback that the coach saw in the hall was criticized before explaining the awkward situation to the principal of the school.

29 f The quarterback that the cheerleaders saw in the hall was criticized before explaining the awkward situation to the principal of the school.

29 g The gymnast that the coaches saw in the hall were criticized before explaining the awkward situation to the principal of the school.

29 h The gymnast that the cheerleader saw in the hall were criticized before explaining the awkward situation to the principal of the school.

30 a The doctor that the dentist advised at the hospital was authorized before filing the insurance forms himself at the office in the hospital.

30 b The doctor that the nutrionist advised at the hospital was authorized before filing the insurance forms himself at the office in the hospital.

30 c The office_assistant that the dentist advised at the hospital was authorized before filing the insurance forms himself at the office in the hospital.

30 d The office_assistant that the nutrionist advised at the hospital was authorized before filing the insurance forms himself at the office in the hospital.

30 e The doctor that the dentist advised at the hospital was authorized before filing the insurance forms at the office in the hospital.

30 f The doctor that the nutrionists advised at the hospital was authorized before filing the insurance forms at the office in the hospital.

30 g The office_assistant that the dentists advised at the hospital were authorized before filing the insurance forms at the office in the hospital.

30 h The office_assistant that the nutrionist advised at the hospital were authorized before filing the insurance forms at the office in the hospital.

31 a The plumber that the inspector contacted at the office was consulted before finishing the entire job himself without help from the young apprentice.

31 b The plumber that the housekeeper contacted at the office was consulted before finishing the entire job himself without help from the young apprentice.

31 c The receptionist that the inspector contacted at the office was consulted before finishing the entire job himself without help from the young apprentice.

31 d The receptionist that the housekeeper contacted at the office was consulted before finishing the entire job himself without help from the young apprentice.

31 e The plumber that the inspector contacted at the office was consulted before finishing the entire job without help from the young apprentice.

31 f The plumber that the housekeepers contacted at the office was consulted before finishing the entire job without help from the young apprentice.

31 g The receptionist that the inspectors contacted at the office were consulted before finishing the entire job without help from the young apprentice.

31 h The receptionist that the housekeeper contacted at the office were consulted before finishing the entire job without help from the young apprentice.

32 a The millionaire that the senator thanked very kindly was interviewed before making a generous contribution himself to the charity for cancer research.

32 b The millionaire that the socialite thanked very kindly was interviewed before making a generous contribution himself to the charity for cancer research.

32 c The countess that the senator thanked very kindly was interviewed before making a generous contribution himself to the charity for cancer research.

32 d The countess that the socialite thanked very kindly was interviewed before making a generous contribution himself to the charity for cancer research.

32 e The millionaire that the senator thanked very kindly was interviewed before making a generous contribution to the charity for cancer research.

32 f The millionaire that the socialites thanked very kindly was interviewed before making a generous contribution to the charity for cancer research.

32 g The countess that the senators thanked very kindly were interviewed before making a generous contribution to the charity for cancer research.

32 h The countess that the socialite thanked very kindly were interviewed before making a generous contribution to the charity for cancer research.

33 a The priest that the schoolboy confronted during class was consulted before writing the lengthy report himself at the new catholic high school.

33 b The priest that the schoolgirl confronted during class was consulted before writing the lengthy report himself at the new catholic high school.

33 c The nun that the schoolboy confronted during class was consulted before writing the lengthy report himself at the new catholic high school.

33 d The nun that the schoolgirl confronted during class was consulted before writing the lengthy report himself at the new catholic high school.

33 e The priest that the schoolboy confronted during class was consulted before writing the lengthy report at the new catholic high school.

33 f The priest that the schoolgirls confronted during class was consulted before writing the lengthy report at the new catholic high school.

33 g The nun that the schoolboys confronted during class were consulted before writing the lengthy report at the new catholic high school.

33 h The nun that the schoolgirl confronted during class were consulted before writing the lengthy report at the new catholic high school.

34 a The senator that the lobbyist emailed about the election was endorsed before revising the annual budget himself over the course of several days.

34 b The senator that the receptionist emailed about the election was endorsed before revising the annual budget himself over the course of several days.

34 c The congresswoman that the lobbyist emailed about the election was endorsed before revising the annual budget himself over the course of several days.

34 d The congresswoman that the receptionist emailed about the election was endorsed before revising the annual budget himself over the course of several days.

34 e The senator that the lobbyist emailed about the election was endorsed before revising the annual budget over the course of several days.

34 f The senator that the receptionists emailed about the election was endorsed before revising the annual budget over the course of several days.

34 g The congresswoman that the lobbyists emailed about the election were endorsed before revising the annual budget over the course of several days.

34 h The congresswoman that the receptionist emailed about the election were endorsed before revising the annual budget over the course of several days.

35 a The legislator that the congressman accused of inappropriate behavior was arrested before explaining the embarrassing situation himself in the letter to the editor.

35 b The legislator that the office_assistant accused of inappropriate behavior was arrested before explaining the embarrassing situation himself in the letter to the editor.

35 c The congresswoman that the congressman accused of inappropriate behavior was arrested before explaining the embarrassing situation himself in the letter to the editor.

35 d The congresswoman that the office_assistant accused of inappropriate behavior was arrested before explaining the embarrassing situation himself in the letter to the editor.

35 e The legislator that the congressman accused of inappropriate behavior was arrested before explaining the embarrassing situation himself in the letter to the editor.

35 f The legislator that the office_assistants accused of inappropriate behavior was arrested before explaining the embarrassing situation himself in the letter to the editor.

35 g The congresswoman that the congressmen accused of inappropriate behavior were arrested before explaining the embarrassing situation himself in the letter to the editor.

35 h The congresswoman that the office_assistant accused of inappropriate behavior were arrested before explaining the embarrassing situation himself in the letter to the editor.

36 a The basketball_player that the coach assisted at the gym was consulted before promoting the new product himself on the billboard along the highway.

36 b The basketball_player that the female_trainer assisted at the gym was consulted before promoting the new product himself on the billboard along the highway.

36 c The softball_player that the coach assisted at the gym was consulted before promoting the new product himself on the billboard along the highway.

36 d The softball_player that the female_trainer assisted at the gym was consulted before promoting the new product himself on the billboard along the highway.

36 e The basketball_player that the coach assisted at the gym was consulted before promoting the new product on the billboard along the highway.

36 f The basketball_player that the female_trainers assisted at the gym was consulted before promoting the new product on the billboard along the highway.

36 g The softball_player that the coaches assisted at the gym were consulted before promoting the new product on the billboard along the highway.

36 h The softball_player that the female_trainer assisted at the gym were consulted before promoting the new product on the billboard along the highway.

37 a The blacksmith that the jester mocked at the festival was arrested after stealing the big roast himself from the owner of the food_stand.

37 b The blacksmith that the maiden mocked at the festival was arrested after stealing the big roast himself from the owner of the food_stand.

37 c The mistress that the jester mocked at the festival was arrested after stealing the big roast himself from the owner of the food_stand.

37 d The mistress that the maiden mocked at the festival was arrested after stealing the big roast himself from the owner of the food_stand.

37 e The blacksmith that the jester mocked at the festival was arrested after stealing the big roast from the owner of the food_stand.

37 f The blacksmith that the maidens mocked at the festival was arrested after stealing the big roast from the owner of the food_stand.

37 g The mistress that the jesters mocked at the festival were arrested after stealing the big roast from the owner of the food_stand.

37 h The mistress that the maiden mocked at the festival were arrested after stealing the big roast from the owner of the food_stand.

38 a The mayor that the protestor denounced at the rally was fired after confirming the spiteful rumors himself in a phone call to the radio.

38 b The mayor that the feminist denounced at the rally was fired after confirming the spiteful rumors himself in a phone call to the radio.

38 c The newswoman that the protestor denounced at the rally was fired after confirming the spiteful rumors himself in a phone call to the radio.

38 d The newswoman that the feminist denounced at the rally was fired after confirming the spiteful rumors himself in a phone call to the radio.

38 e The mayor that the protestor denounced at the rally was fired after confirming the spiteful rumors in a phone call to the radio.

38 f The mayor that the feminists denounced at the rally was fired after confirming the spiteful rumors in a phone call to the radio.

38 g The newswoman that the protestors denounced at the rally were fired after confirming the spiteful rumors in a phone call to the radio.

38 h The newswoman that the feminist denounced at the rally were fired after confirming the spiteful rumors in a phone call to the radio.

39 a The boxer that the sports_agent interviewed on television was congratulated after promoting the training device himself during the charity event for kids.

39 b The boxer that the female_reporter interviewed on television was congratulated after promoting the training device himself during the charity event for kids.

39 c The gymnast that the sports_agent interviewed on television was congratulated after promoting the training device himself during the charity event for kids.

39 d The gymnast that the female_reporter interviewed on television was congratulated after promoting the training device himself during the charity event for kids.

39 e The boxer that the sports_agent interviewed on television was congratulated after promoting the training device during the charity event for kids.

39 f The boxer that the female_reporters interviewed on television was congratulated after promoting the training device during the charity event for kids.

39 g The gymnast that the sports_agents interviewed on television were congratulated after promoting the training device during the charity event for kids.

39 h The gymnast that the female_reporter interviewed on television were congratulated after promoting the training device during the charity event for kids.

40 a The businessman that the stockbroker consulted at work was promoted after reviewing the annual report himself at the meeting for the corporation.

40 b The businessman that the saleswoman consulted at work was promoted after reviewing the annual report himself at the meeting for the corporation.

40 c The female_intern that the stockbroker consulted at work was promoted after reviewing the annual report himself at the meeting for the corporation.

40 d The female_intern that the saleswoman consulted at work was promoted after reviewing the annual report himself at the meeting for the corporation.

40 e The businessman that the stockbroker consulted at work was promoted after reviewing the annual report at the meeting for the corporation.

40 f The businessman that the saleswomen consulted at work was promoted after reviewing the annual report at the meeting for the corporation.

40 g The female_intern that the stockbrokers consulted at work were promoted after reviewing the annual report at the meeting for the corporation.

40 h The female_intern that the saleswoman consulted at work were promoted after reviewing the annual report at the meeting for the corporation.

41 a The elderly_man that the doctor treated at the hospital was released after reading the medical report himself in the very crowded examination room.

41 b The elderly_man that the nurse treated at the hospital was released after reading the medical report himself in the very crowded examination room.

41 c The elderly_woman that the doctor treated at the hospital was released after reading the medical report himself in the very crowded examination room.

41 d The elderly_woman that the nurse treated at the hospital was released after reading the medical report himself in the very crowded examination room.

41 e The elderly_man that the doctor treated at the hospital was released after reading the medical report in the very crowded examination room.

41 f The elderly_man that the nurses treated at the hospital was released after reading the medical report in the very crowded examination room.

41 g The elderly_woman that the doctors treated at the hospital were released after reading the medical report in the very crowded examination room.

41 h The elderly_woman that the nurse treated at the hospital were released after reading the medical report in the very crowded examination room.

42 a The businessman that the executive met in college was congratulated after completing the difficult task himself without any assistance from the staff.

42 b The businessman that the receptionist met in college was congratulated after completing the difficult task himself without any assistance from the staff.

42 c The file_clerk that the executive met in college was congratulated after completing the difficult task himself without any assistance from the staff.

42 d The file_clerk that the receptionist met in college was congratulated after completing the difficult task himself without any assistance from the staff.

42 e The businessman that the executive met in college was congratulated after completing the difficult task without any assistance from the staff.

42 f The businessman that the receptionists met in college was congratulated after completing the difficult task without any assistance from the staff.

42 g The file_clerk that the executives met in college were congratulated after completing the difficult task without any assistance from the staff.

42 h The file_clerk that the receptionist met in college were congratulated after completing the difficult task without any assistance from the staff.

43 a The co-pilot that the body_guard greeted on the plane was commended after landing the damaged plane himself in the empty field of grass.

43 b The co-pilot that the stewardess greeted on the plane was commended after landing the damaged plane himself in the empty field of grass.

43 c The countess that the body_guard greeted on the plane was commended after landing the damaged plane himself in the empty field of grass.

43 d The countess that the stewardess greeted on the plane was commended after landing the damaged plane himself in the empty field of grass.

43 e The co-pilot that the body_guard greeted on the plane was commended after landing the damaged plane in the empty field of grass.

43 f The co-pilot that the stewardesses greeted on the plane was commended after landing the damaged plane in the empty field of grass.

43 g The countess that the body_guards greeted on the plane were commended after landing the damaged plane in the empty field of grass.

43 h The countess that the stewardess greeted on the plane were commended after landing the damaged plane in the empty field of grass.

44 a The carpenter that the mechanic recommended for the job was hired after confirming the estimated cost himself during the interview over the phone.

44 b The carpenter that the kindergarten_teacher recommended for the job was hired after confirming the estimated cost himself during the interview over the phone.

44 c The cleaning_lady that the mechanic recommended for the job was hired after confirming the estimated cost himself during the interview over the phone.

44 d The cleaning_lady that the kindergarten_teacher recommended for the job was hired after confirming the estimated cost himself during the interview over the phone.

44 e The carpenter that the mechanic recommended for the job was hired after confirming the estimated cost during the interview over the phone.

44 f The carpenter that the kindergarten_teachers recommended for the job was hired after confirming the estimated cost during the interview over the phone.

44 g The cleaning_lady that the mechanics recommended for the job were hired after confirming the estimated cost during the interview over the phone.

44 h The cleaning_lady that the kindergarten_teacher recommended for the job were hired after confirming the estimated cost during the interview over the phone.

45 a The technician that the computer_programmer introduced to the office was complimented after installing the new software himself in the computers for the executives.

45 b The technician that the saleswoman introduced to the office was complimented after installing the new software himself in the computers for the executives.

45 c The telephonist that the computer_programmer introduced to the office was complimented after installing the new software himself in the computers for the executives.

45 d The telephonist that the saleswoman introduced to the office was complimented after installing the new software himself in the computers for the executives.

45 e The technician that the computer_programmer introduced to the office was complimented after installing the new software in the computers for the executives.

45 f The technician that the saleswomen introduced to the office was complimented after installing the new software in the computers for the executives.

45 g The telephonist that the computer_programmers introduced to the office were complimented after installing the new software in the computers for the executives.

45 h The telephonist that the saleswoman introduced to the office were complimented after installing the new software in the computers for the executives.

46 a The judge that the lawyer referenced in the letter was dismissed after revealing the test results himself during the very controversial court case.

46 b The judge that the female_intern referenced in the letter was dismissed after revealing the test results himself during the very controversial court case.

46 c The newswoman that the lawyer referenced in the letter was dismissed after revealing the test results himself during the very controversial court case.

46 d The newswoman that the female_intern referenced in the letter was dismissed after revealing the test results himself during the very controversial court case.

46 e The judge that the lawyer referenced in the letter was dismissed after revealing the test results during the very controversial court case.

46 f The judge that the female_interns referenced in the letter was dismissed after revealing the test results during the very controversial court case.

46 g The newswoman that the lawyers referenced in the letter were dismissed after revealing the test results during the very controversial court case.

46 h The newswoman that the female_intern referenced in the letter were dismissed after revealing the test results during the very controversial court case.

47 a The sheriff that the newsman congratulated at the press_conference was interviewed after solving the mysterious crime himself in just a few short days

47 b The sheriff that the female_paralegal congratulated at the press_conference was interviewed after solving the mysterious crime himself in just a few short days

47 c The policewoman that the newsman congratulated at the press_conference was interviewed after solving the mysterious crime himself in just a few short days

47 d The policewoman that the female_paralegal congratulated at the press_conference was interviewed after solving the mysterious crime himself in just a few short days

47 e The sheriff that the newsman congratulated at the press_conference was interviewed after solving the mysterious crime in just a few short days

47 f The sheriff that the female_paralegals congratulated at the press_conference was interviewed after solving the mysterious crime in just a few short days

47 g The policewoman that the newsmen congratulated at the press_conference were interviewed after solving the mysterious crime in just a few short days

47 h The policewoman that the female_paralegal congratulated at the press_conference were interviewed after solving the mysterious crime in just a few short days

48 a The mechanic that the car_salesman met at the car_dealership was fired after driving the expensive sports_car himself without permission from the new boss.

48 b The mechanic that the salesgirl met at the car_dealership was fired after driving the expensive sports_car himself without permission from the new boss.

48 c The telephonist that the car_salesman met at the car_dealership was fired after driving the expensive sports_car himself without permission from the new boss.

48 d The telephonist that the salesgirl met at the car_dealership was fired after driving the expensive sports_car himself without permission from the new boss.

48 e The mechanic that the car_salesman met at the car_dealership was fired after driving the expensive sports_car without permission from the new boss.

48 f The mechanic that the salesgirls met at the car_dealership was fired after driving the expensive sports_car without permission from the new boss.

48 g The telephonist that the car_salesmen met at the car_dealership were fired after driving the expensive sports_car without permission from the new boss.

48 h The telephonist that the salesgirl met at the car_dealership were fired after driving the expensive sports_car without permission from the new boss.
